# Shift workers’ experiences and views of sleep disturbance, fatigue and healthy behaviors: a systematic review and qualitative evidence synthesis

**DOI:** 10.5271/sjweh.4223

**Published:** 2025-07-01

**Authors:** Jack S Benton, Charlotte L Lee, Hannah A Long, Thavapriya Sugavanam, Leah Holmes, Annie Keane, Neal Thurley, Simon Kyle, David Ray, David P French

**Affiliations:** 1 Manchester Centre for Health Psychology, Division of Psychology & Mental Health, School of Health Sciences, Faculty of Biology, Medicine and Health, University of Manchester, Manchester, UK.; 2 Sir Jules Thorn Sleep and Circadian Neuroscience Institute, Nuffield Department of Clinical Neuroscience, University of Oxford, Oxford, UK.; 3 Division of Nursing, Midwifery and Social Work, School of Health Sciences, Faculty of Biology, Medicine and Health, University of Manchester, Manchester, UK.; 4 NIHR Oxford Health Biomedical Research Centre, and NIHR Oxford Biomedical Research Centre, John Radcliffe Hospital, Oxford, UK.; 5 Oxford Centre for Diabetes, Endocrinology and Metabolism, and Oxford Kavli Centre for Nanoscience Discovery, University of Oxford, Oxford, UK.; 6 Vocal, Manchester University NHS Foundation Trust, Manchester, UK.; 7 Bodleian Health Care Libraries, Oxford University Hospitals NHS Foundation Trust, Oxford, UK.

**Keywords:** insomnia, meta-synthesis, occupational health, qualitative research, shift work disorder, work

## Abstract

**Objective:**

Shift work is common across most societies but poses significant risks to the health of shift workers. In part, this risk is due to the disruption of healthy sleep-wake schedules. This systematic review identified qualitative research on shift workers’ experiences of sleep disturbance, fatigue and healthy behaviors.

**Methods:**

We conducted a systematic search of four databases (CINAHL, EMBASE, MEDLINE, PsycINFO) and identified 28 eligible studies involving 1519 participants. We appraised the studies using an adapted Critical Appraisal Skills Programme (CASP) checklist, and confidence in the review findings was formally assessed using the Grading of Recommendations Assessment, Development and Evaluation-Confidence in the Evidence from Reviews of Qualitative research (GRADE‐CERQual) approach. Data were thematically synthesized.

**Results:**

Three analytical themes were generated. ‘Inevitability of fatigue and tiredness’ outlines how shift workers experience a culture where they feel “peer pressure to soldier through” their shifts regardless of fatigue. ‘Balancing sleep needs with competing responsibilities’ highlights how shift workers struggle to balance the need for daytime sleep with family, leisure, and work responsibilities, often prioritizing family needs over their own sleep. ‘Obstacles to engaging in healthy behaviors’ describes how shift workers often know which actions would benefit their health and reduce fatigue but find it challenging to translate this knowledge into behavior due to fatiguing and stressful work environments. For the purposes of the GRADE-CERQual assessment, short summary statements were developed to describe 22 review findings: there was moderate or high confidence in all but one of these findings.

**Conclusion:**

This review suggests that sleep education alone is unlikely to be effective. Interventions should focus on helping shift workers self-regulate their behaviors, thoughts, and emotions to better manage sleep and fatigue.

Shift work is typically defined as any work schedule outside standard daytime hours (approximately 08:00–18:00 hours) (1), including early morning, evening, night, and rotating shifts. It is common across many industries including healthcare, emergency services, transportation, and aviation. Around 20% of workers are involved in some form of shift work (2). Shift work disrupts the alignment between the external environment and the body’s internally-driven circadian rhythm (body clock), leading to sleep difficulties and health issues (3). Shift workers also face unpredictable employment, on-call duties, and long hours, which can negatively affect sleep, work performance, and wellbeing (4–7).

Although pharmacological interventions (eg, melatonin) are used to address shift work-related sleep disturbance, they may produce adverse side effects, including headaches, nausea and insomnia (8). Non-pharmacological interventions, such as light therapy, strategic napping, and cognitive-behavioral techniques, offer alternative approaches. However, systematic reviews of non-pharmacological interventions have yielded mixed findings, and the quality of studies vary (9–11).

Understanding shift workers’ experiences is vital for developing effective and acceptable interventions. A recent systematic review of qualitative studies provided evidence on licensed nurses working night shifts (12), which highlighted the challenges of sleep deprivation and the need for organizational support to improve nurse and patient safety. However, it excluded other types of healthcare professionals (eg, doctors) and non-healthcare shift workers. It also excluded studies deemed to be of lower quality, a practice explicitly discouraged by Cochrane guidance, due to quality cutoffs being arbitrary and hence not methodologically defensible (13). Other qualitative systematic reviews have examined experiences of shift workers (14–16), but none have focused on understanding shift workers’ experiences of sleep disturbance, fatigue and healthy behaviors. To address this gap, the present systematic review aimed to: (i) systematically identify and appraise qualitative and mixed-methods studies of shift workers’ experiences and views of sleep disturbance, fatigue and healthy behaviors; (ii) use thematic synthesis to analyze and synthesize findings of relevant qualitative and mixed-methods studies; and (iii) identify directions for future research to improve the acceptability of sleep interventions for shift workers.

## Methods

The protocol for this qualitative systematic review was registered on PROSPERO (CRD42023468410) (17). This review is reported in accordance with the Preferred Reporting Items for Systematic Reviews and Meta-Analyses (PRISMA) (18) (supplementary material, www.sjweh.fi/article/4223, appendix 1) and Enhancing Transparency in Reporting the Synthesis of Qualitative Research (ENTREQ) (19) guidelines (supplementary appendix 2).

### Eligibility criteria

*Types of studies.* Full details of eligibility criteria are provided in table 1. We included primary studies that used qualitative methods for both data collection and analysis. Mixed-methods studies were included if qualitative data could be extracted. Only peer-reviewed studies published in English were included due to practical constraints.

*Topic of interest.* We included studies on adults (aged ≥18 years) involved in shift work (ie, early morning, evening, night, or rotating shifts) in any occupation. Studies with both shift and non-shift workers were included only if shift worker findings could be extracted. Phenomena of interest were shift workers’ experiences of sleep disturbance, fatigue and healthy behaviors. Studies exploring health behaviors (eg, diet, physical activity) were included only if these health behaviors were explored as precipitating or perpetuating factors of sleep.

**Table 1:** Eligibility criteria.

**Inclusion criteria**
(a) Qualitative methods for data collection (eg, individual interviews, focus group discussions, open-ended survey questions) and analysis (eg, thematic analysis, framework analysis, grounded theory).
(b) Mixed methods studies, provided that there was a substantial qualitative element illustrated by author interpretation and participant quotes, and it was possible to extract the data that were collected and analyzed using qualitative methods.
(c) Adults (aged 18+ years) working shift work (ie, early morning, evening, night, or rotating shifts) in any occupation.
(d) Mixed samples of shift workers and non-shift workers were only included if it was possible to separately identify those findings related to the shift workers.
(e) Studies that included a focus on shift workers’ experiences and views of sleep disturbance and its impacts in relation to any occupation of shift work, regardless of whether this was in relation to an intervention or not.
(f) Studies that explored shift workers’ experiences of health behaviors (eg, diet, physical activity, caffeine use) were only included if these health behaviors were explored as precipitating or perpetuating factors of sleep.
(g) Published in English.
**Exclusion criteria**
(a) Quantitative only studies (eg, open-ended survey questions where the response data are analyzed using descriptive statistics only).
(b) Studies could not be separated in inclusion criterion (d).
(c) Grey or unpublished literature.
(d) Review articles; Comments; Conference proceedings; Study protocols.
(e) Full text was not available.

### Search strategy

The following electronic databases were searched up to 1 August 2023: CINAHL, EMBASE, MEDLINE, PsycINFO. We selected medical subject headings (MeSH terms) and free-text terms (eg keywords in article titles and abstracts) for: shift work, sleep, and qualitative terms. Full details on the search strategy are provided in supplementary appendix 3. Results were exported to Covidence (20) where duplicates were removed. Reference lists were checked for those records that matched our inclusion criteria. We also employed forwards and backwards citation tracking.

### Selection of studies

The first author independently screened the titles and abstracts of the identified records to evaluate eligibility, and a second reviewer independently screened 10% (N=448) of these. Two reviewers then read the full text of all potentially eligible articles and assessed these against the eligibility criteria. Disagreements were resolved through discussion between three reviewers.

### Data extraction

Two reviewers developed and piloted a data extraction form. Extracted data included: author(s) and date of publication; research aims; study setting; sample characteristics; data collection and analysis methods; and all reported findings. Data extracted for synthesis included all direct quotations from participants (first-order constructs) and the authors’ interpretations of their study findings (second-order constructs). We extracted all text under the Results/Findings sections and any findings in the abstract for synthesis (21). One reviewer independently extracted the data from all included articles and another double-coded a random sample of 10% (N=3) of included articles.

### Quality appraisal

One reviewer independently assessed study quality using a modified version of the Critical Appraisal Skills Programme (CASP) qualitative checklist tool (22). This tool was chosen to assess study validity, rigor, and utility of findings, including quality appraisal of the ontological and epistemological underpinnings. A second reviewer independently assessed the quality of two studies and a third assessed one study. Disagreements were resolved through discussion. In line with Cochrane guidance (13), we did not exclude studies based on our assessment of quality. Instead, we used the quality appraisal to assess confidence in the findings (see the section 'Assessing confidence in the review findings').

### Data synthesis

We used Thomas & Harden's thematic synthesis method (21), which allowed us to stay true to the included studies while adding our interpretations for new insights. The present approach to inquiry was informed by dialectical pluralism, which enables integration of qualitative research undertaken from multiple perspectives (23).

Data were analyzed in NVivo version 12 (International Q. NVivo qualitative data analysis software, 2018). The first reviewer performed inductive line-by-line coding by attaching labels to meaningful units of text in regular discussion with two other reviewers. Participant quotations were coded first, then the authors’ narratives.

Second, codes were organized into six descriptive themes based on similarities and differences. A draft summary of the descriptive themes was produced by the first reviewer and discussed with four other reviewers. Descriptive themes were also discussed with our Patient and Public Involvement, Engagement and Participation (PPIEP) group of healthcare shift workers to ensure the findings aligned with their lived experiences. In a facilitated online discussion, PPIEP members’ experiences closely reflected the descriptive themes, with worry emerging as a particularly prominent issue across all participants. This discussion reinforced the salience of worry in the analysis, leading us to emphasize this theme in our reporting. Finally, the first reviewer interpreted, analyzed, and conceptually synthesized the descriptive themes into analytical themes. This was done by considering the conceptual links between descriptive themes to develop new insights and concepts. Preliminary analytical themes were discussed with six other reviewers on multiple occasions to finalize the most appropriate thematic structure and discuss the expression of the synthesis.

### Assessing confidence in the review findings

We used the GRADE-CERQual (Grading of Recommendations Assessment, Development and Evaluation-Confidence in the Evidence from Reviews of Qualitative research) approach to assess confidence in each review finding (24). According to GRADE-CERQual guidance, confidence assessments should be applied to specific review findings rather than to overarching themes or brief theme/code labels (25). In our review, thematic synthesis and GRADE-CERQual served different purposes: thematic synthesis structured the interpretation of data, while GRADE-CERQual assessed confidence in specific findings. We first conducted a thematic synthesis, identifying six descriptive themes from coded data, which were then synthesized into three overarching analytic themes. To align with GRADE-CERQual guidance, we developed 22 review findings based on the thematic synthesis, primarily reflecting the descriptive themes. Two reviewers developed and agreed on these statements before conducting GRADE-CERQual assessments.

The first reviewer applied the GRADE-CERQual tool to each review finding to assess confidence across four components: (i) methodological limitations of the included studies, (ii) coherence of the finding, (iii) adequacy of the data supporting the finding, and (iv) relevance of the included studies to the review question. Overall confidence levels were deemed as high, moderate, low, or very low. Two reviewers discussed and agreed on the assessments.

## Results

The electronic database and hand searches identified 7181 records (4478 records after duplicates removed). Two reviewers screened full texts of 85 articles (93% agreement; 0.71 kappa, indicating substantial agreement) (26), from which 28 were included (27–54) (figure 1). The 57 articles excluded at full-text screening stage (with reasons for exclusion) are reported in supplementary appendix 4.

**Figure:**
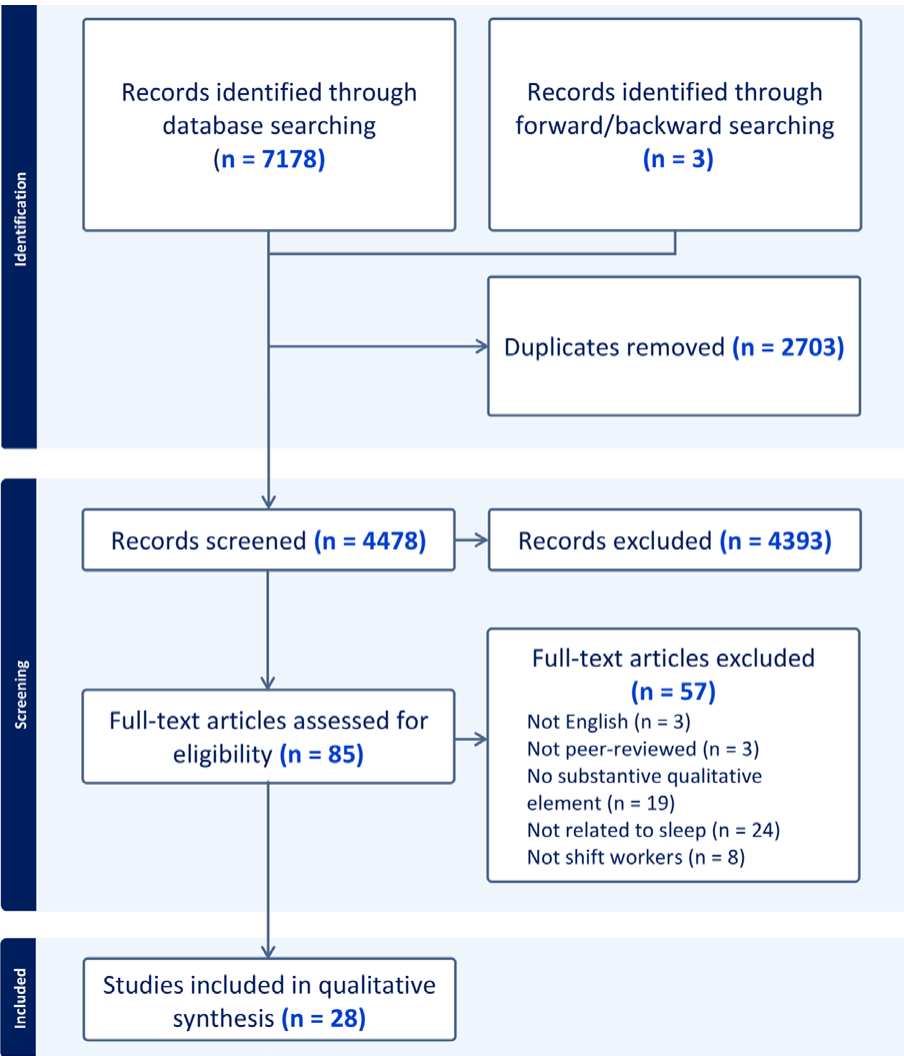
**Figure 1.** PRISMA flow chart

### Description of the studies

Study characteristics are summarized in table 2. All 28 studies were published in English. Most studies were conducted in the United States (US) (N=10), Australia (N=6); and the United Kingdom (UK) (N=5). The studies included a total of 1519 participants. Studies included healthcare shift workers (N=19), non-healthcare shift workers (bus drivers, flight attendants, firefighters, frontline tunnelling workers, or night-time economy workers) (N=6), and a mixed sample of healthcare and non-healthcare shift workers (N=3). Participants worked a range of shifts, which mostly included night (N=11), rotating (N=8) (ie, shifts that alternate between day and night), and day shifts (N=7).

**Table 2:** Main characteristics of included studies. [F=female; FG=focus groups; M=males; NR=not reported.]

Author (reference)	Country	Study focus	Participant characteristics				Qualitative data collection methods	Qualitative data analysis
			Sample ^1^	Demographics ^2^	Occupation	Shift type		
Aemmi et al (27)	Iran	Sleep management process in shift-working nurses	N=18	Age range 25–45; F:15 (83.3%), M:3 (16.7%); Ethnicity NR	Nurses	Rotating	Semi-structured interviews	Constant comparative analysis (grounded theory)
Arbour et al (28)	United States	Midwives’ experiences related to sleep and sleep deprivation	N=268	Age range 26–75 (mean age 52); F: 264 (98.5%); 248 White (92.4%)	Midwives	NR	Open-ended survey question (mixed-methods study)	Inductive content analysis
Bushell (29)	United Kingdom	Sleep deprivation amongst migrant workers in NE England’s night-time economy	Interviews: N=268 Vignettes: N=35	NR	Night-time economy workers ^3^	NR	(1) unstructured interviews (2) short vignettes	Thematic analysis
Centofanti et al (30)	Australia	Nurses and midwives use of napping and caffeine countermeasures	N=22	Age NR; F:19 (86.4%), M: 3 (13.6%); Ethnicity NR	Nurses and midwives	Forward rotating	Interviews (mixed-methods study)	Thematic analysis
Epstein et al (31)	Sweden	Newly graduated nurses’ strategies and experiences of sleep problems and fatigue	N=11	Age range 22–51; Gender/sex NR; Ethnicity NR	Nurses (newly graduated)	Morning, evening, night	Semi-structured interviews	Inductive content analysis
Fallis et al (32)	Canada	Nurses’ perceptions, experiences, barriers, and safety issues related to napping during night shift	N=13	Age range 18–65 (15% 18–30, 54% 31–50, 31% 51–65); F: 11 (84.6%), M: 2 (15.4%); Ethnicity NR	Nurses (critical care and emergency department)	Day, evening, night	Semi-structured interviews	Constant comparative analysis
Gallew & Mu (33)	United States	Experiences and strategies of night shift nurses for sleep, social & domestic interactions, and work performance	N=11	Age range 20–59 (64% 20–29, 36% 40–59); Gender/sex NR; Ethnicity NR	Nurses (nursery and birthing centers)	Night	Semi-structured interviews	Thematic analysis
Geiger-Brown et al (34)	United States	Nurses’ perceptions about napping following implementation of a napping initiative	N=98	NR	Registered nurses	Night	Open-ended survey question (mixed- methods study)	Content analysis
Gifkins et al (35)	Australia	Food choices and eating patterns of nurses exposed to different shift work lengths	N=12	Age NR; F:12 (100%); Ethnicity NR	Nurses	NR	Semi-structured interviews	Thematic analysis
Groves et al (36)	United States	How hospital nurses manage fatigue, and barriers and strategies at home and work	N=41	Age range 25–49 (mean age 35.4); F: 39 (95.1%), M: 2 (4.9%); Ethnicity NR	Registered nurses	Day, rotating, night	Semi-structured interviews	Content analysis
Jay et al (37)	Australia	Women’s experiences of being on-call in emergency services and their coping strategies	Interviews: N=18 FG: N=6	Age NR; 24 F (100%); Ethnicity NR	Emergency service personnel	NR	(1) semi-structured interviews (2) a semi-structured FG (mixed-methods study)	Thematic analysis
Klinefelter et al (38)	United States	Emergency physicians’ perceptions and experiences of fatigue	Interviews: N=15 FG: N=5	Age range 30–63 (mean age 42.6); F: 11 (55%), M: 9 (45%); 15 White (75%), 4 ≥2 races (20%), Asian (5%);	Emergency physicians	NR	(1) semi-structured interviews (2) semi-structured FG	Grounded theory
Lowson & Arber (39)	United Kingdom	Perspectives of night-working women nurses and their families regarding practices surrounding their night work	N=20	Age NR; F: 20 (100%); Ethnicity NR	Nurses	Early, late, rotating, night	(1) joint qualitative interviews (nurses & partners) (2) audio sleep diaries (3) a separate qualitative interview (post audio sleep diaries)	Constant comparative analysis
Maynard et al (40)	United Kingdom	City bus drivers’ experiences of fatigue, and its influence on driving and management strategies	N=65	Age range 25–64 (16.9% 25–34, 21.5% 35–44, 35.4% 45–54, 26.2% 55–64); F: 13 (20%), M: 52 (80%); Ethnicity NR	Bus drivers	Early, middle, late, night	Semi-structured FG	Thematic analysis
Maynard et al (41)	United Kingdom	Tunnellers’ and their managers’ opinions on the influence of fatigue in tunnelling and its management	N=42	Age range 18–64 (3% 18–24, 23% 25–34, 20% 35–44, 31% 45–54, 23% 55–64); Gender/sex NR; Ethnicity NR	Frontline tunnelling workers	Day, evening, night, backward rotating	Semi-structured FG	Thematic analysis
McIntosh et al (42)	NR ^4^	Barriers to the healthy eating behaviors of shift workers	N=144	NR	Mixed (nurses, truck drivers, fly-in/-out work workers)	NR ^4^	Qualitative analysis of social media data	Thematic content analysis
Nea et al (43)	Ireland & Northern Ireland	The shift work experience and its impact on dietary and lifestyle practices among shift workers	N=109	Age range 18–64 (16.5% 18–25, 31.2% 26–35, 22.9% 36–45, 24.8% 46–55, 3.7% 56–64); F: 44 (40.4%), M: 65 (59.6%); Ethnicity NR	Mixed (accommodation and food services, health and social care, and manufacturing/ industry)	Day, night, rotating	Semi-structured FG	Inductive thematic analysis
Nyberg et al (44)	Nordic countries	How work organization, time, and place affect flight attendants’ food and meal situations	N=14	Age range 25–54; F: 10 (71.4%), M: 4 (28.6%); Ethnicity NR	Flight attendants	Short- and long-haul flights	Semi-structured interviews	Thematic analysis
Paterson et al (45)	Australia	Factors paramedics recognize as contributors to fatigue	N=49	Mean age 38; F: 12 (24.5%), M: 37 (75.5%); Ethnicity NR	Paramedics	NR	An open-ended survey question	Inductive content analysis
Persson et al (46)	Sweden	Situations that affect diet and exercise habits among night-shift nurses	N=27	Age range 25–63 (22.2% 25–34, 29.6% 35–44, 22.2% 45–54, 25.9% 55–63); F: 25 (92.6%), M: 2 (7.4%); Ethnicity NR	Enrolled and registered nurses	Night	Semi-structured interviews	Critical Incident Technique
Pilkington-Cheney et al (47)	United Kingdom	How city bus drivers manage and counteract sleepiness	N=62	Age range 25–65 (16.9% 25–34, 21% 35–44, 35.5% 45–54, 26.2% 54–65); F: 13 (21%), M: 49 (79%); Ethnicity NR	Bus drivers	Day, night	Semi-structured FG	Inductive thematic analysis
Reynolds et al (48)	Australia	Experience of sleep disorder diagnosis and treatment in shift workers and explore informed solutions to improve health service access	N=16	Mean age 48.5; F: 7 (38.9%), M: 8 (44.4%), 1 non–binary/ intersex/ other (5.6%); Ethnicity NR	Mixed (nursing and hospital administration support, aged care support, rail drivers and engineering/ support, telecommunications, security services, hospitality and retail workers)	Rotating, fixed, on call, irregular	Semi-structured interviews	Reflexive thematic analysis and patient journey mapping
Smith et al (49)	United States	Night shift nurses’ perceptions of drowsy driving, countermeasures, and interventions for drowsiness	N=30	Age range 22–64 (mean age 36.1); F: 20 (66.6%), M: 10 (33.3%); 15 Asian (50%), 8 Black (26.67%), 5 White (16.67%), 1 multiple (3.33%), 1 other (3.33%)	Nurses	Night	Semi-structured interviews	Inductive thematic analysis
Smith-Miller et al (50)	United States	Barriers and facilitators in implementing a fatigue management plan and its effects on unit culture and individual experiences	NR	NR	Nurses	Day, night	FG (mixed-methods study)	Comparative analysis
Steege and Rainbow (51)	United States	Barriers and facilitators in the hospital nursing system affecting coping and fatigue	N=22	Age range 23–55 (mean age 30.73); F: 19 (86.4%), M: 3 (13.6%); Ethnicity NR	Registered nurses	Day, night, rotating	Semi-structured interviews	Directed content analysis
Stimpfel et al (52)	United States	How nurses perceive work organization factors that impact their performance	N=23	Age range 18–49; F: 17 (73.9%), M: 2 (8.7%); 16 White (68.4%), 2 African American/ Black (10.5%), 2 Asian (10.5%), 1 Latino or Hispanic (5.3%), 1 Mixed race (5.3%)	Registered nurses	Day, evening, night	Asynchronous virtual FG (i.e., threaded discussion boards)	Directed content analysis
Torquati et al (53)	Australia	Barriers and enablers of healthy diet and physical activity behaviors in nurses	N=17	Age range 25–59 (mean age 39.5); F: 14 (82.4%), M: 3 (17.6%) Ethnicity NR	Registered nurses and clinical nurses	Day, night	Semi-structured FG	Thematic analysis using a realist approach
Watkins et al (54)	United States	How family dynamics and fire department’s shift schedule influence’ prioritization of sleep and recovery at home	N=38	Age range 25–45+ (14% 25–34, 55% 35–44, 29% ≥45 Majority M; Ethnicity NR	Firefighters	24–48 ^5^	Semi-structured FG	Grounded theory

^1^ The sample size only includes shift workers, rather than any other participants (eg, family members, managers) ^2^ Age (years), gender/sex, and ethnicity. ^3^ The sample includes food take-away workers, security staff and taxi drivers. ^4^ The study examined Facebook comments, which means that the geographical origins of the participants and their shift type were not apparent. ^5^ A 24-48 shift schedule consists of a 3-day cycle where each team works one 24-hour shift followed by two consecutive days (48 hours) off duty.

### Quality appraisal results

The CASP quality appraisal results are shown in table 3. Only two studies adequately reported the theoretical underpinnings of the research, and only four studies sufficiently considered the influence of the researcher on the study design and results. Nearly one-third of studies (N=8) did not clearly report their recruitment strategy. The quality of data analysis was mixed: thirteen studies were rated as significantly rigorous, seven as somewhat rigorous, six as lower quality, and two lacked sufficient information.

**Table 3:** CASP quality appraisal results.

Authors (reference)	Modified CASP question									
	1. Was there a clear statement of the aims of the research?	2. Is a qualitative methodology appropriate?	3. Was the research design appropriate to address the aims of the research?	4. Are the study’s theoretical underpinnings clear, consistent and conceptually coherent?	5. Was the recruitment strategy appropriate to the aims of the research?	6. Was the data collected in a way that addressed the research issue?	7. Has the relationship between researcher and participants been adequately considered?	8. Have ethical issues been taken into consideration?	9. Was the data analysis sufficiently rigorous?	11. Is there a clear statement of findings?
Aemmi et al (27)	Yes	Yes	Yes	Somewhat	Yes	Yes	Somewhat	Somewhat	Yes	Somewhat
Arbour et al (28)	Yes	Yes	No	No	No	No	No	Yes	No	No
Bushell (29)	Yes	Yes	Yes	Yes	Yes	Can’t tell	No	Somewhat	Can’t tell	Somewhat
Centofanti et al (3)	Yes	Somewhat	Somewhat	No	Can’t tell	Can’t tell	No	Somewhat	No	Somewhat
Epstein et al (31)	Yes	Yes	Yes	No	Yes	Yes	Yes	Yes	Yes	Yes
Fallis et al (32)	Yes	Yes	Yes	No	Yes	Yes	Yes	Somewhat	Yes	Yes
Gallew and Mu (33)	Yes	Yes	Yes	No	Yes	Yes	Somewhat	No	Somewhat	Somewhat
Geiger-Brown et al (34)	Somewhat	Yes	No	No	Can’t tell	No	Yes	Yes	Somewhat	Yes
Gifkins et al (35)	Yes	Yes	Yes	Somewhat	Yes	Can’t tell	No	Somewhat	Can’t tell	Yes
Groves et al (36)	Yes	Yes	Yes	No	Yes	Yes	Somewhat	Yes	Yes	Yes
Jay et al (37)	Yes	Yes	Yes	No	Can’t tell	Somewhat	No	Somewhat	No	No
Klinefelter et al (38)	Yes	Yes	Yes	No	Yes	Yes	No	Somewhat	No	No
Lowson and Arber (39)	Yes	Yes	Yes	Somewhat	Can’t tell	Yes	No	Somewhat	Somewhat	Yes
Maynard et al (40)	Yes	Yes	Yes	No	Yes	Yes	No	Somewhat	Yes	Yes
Maynard et al (41)	Yes	Yes	Yes	No	Can’t tell	Somewhat	No	No	Yes	Yes
McIntosh et al (42)	Yes	Yes	Yes	No	Yes	Yes	Yes	Somewhat	Yes	Yes
Nea et al (43)	Yes	Yes	Yes	No	Yes	Somewhat	No	Somewhat	No	Somewhat
Nyberg et al (44)	Yes	Yes	Yes	No	Yes	Yes	Somewhat	Somewhat	Yes	Somewhat
Paterson et al (45)	Yes	Yes	No	No	No	No	No	No	Somewhat	Yes
Persson et al (46)	Yes	Yes	Yes	No	Can’t tell	Yes	No	Somewhat	Somewhat	Somewhat
Pilkington-Cheney et al (47)	Yes	Yes	Yes	No	Can’t tell	Yes	Somewhat	Somewhat	Somewhat	Yes
Reynolds et al (48)	Yes	Yes	Yes	Yes	Yes	Yes	No	Somewhat	Yes	Yes
Smith et al (49)	Yes	Yes	Yes	No	Somewhat	Yes	Somewhat	Somewhat	Somewhat	Somewhat
Smith-Miller et al (50)	Yes	Yes	Yes	No	Can’t tell	Somewhat	Somewhat	No	No	No
Steege and Rainbow (51)	Yes	Yes	Yes	Somewhat	Yes	Yes	No	Somewhat	Yes	Yes
Stimpfel et al (52)	Yes	Yes	Yes	Somewhat	Yes	Yes	No	No	Yes	Yes
Torquati et al (53)	Yes	Yes	Yes	Somewhat	Yes	Yes	No	Yes	Yes	Yes
Watkins et al (54)	Yes	Yes	Somewhat	No	Yes	Yes	No	Somewhat	Yes	Yes

### Thematic synthesis results

Six descriptive themes were generated (see supplementary appendix 5) and developed into three analytical themes: (i) inevitability of fatigue and tiredness, (ii) balancing sleep needs with competing responsibilities, and (iii) obstacles to engaging in healthy behaviors. Direct quotes from study participants are presented in italics within quotation marks, while quotes from study authors are shown in italics without quotation marks.

### Inevitability of fatigue and tiredness

Fatigue and tiredness were used interchangeably and commonly reported as a concern amongst shift workers. Fatigue was often perceived to be an inevitable experience of shift work: “*I think fatigue is inevitable in emergency medicine, regardless of where you work. It’s just the nature of the beast.*” (38). For many workers, fatigue seemed unavoidable due to (i) difficulties getting enough quality sleep when working non-standard hours, and (ii) task-related fatigue (41) associated with characteristics of shift work, including long hours, heavy workloads, insufficient breaks, and staff shortages. Therefore, they often perceived fatigue as “*part of the job*” (51): “*I’d have thought everybody [experienced fatigue], everybody. If they said they haven’t, I think they’d be lying.*” (40).

Shift workers recognized the consequences of fatigue on physical and mental health. They often reported excessive sleepiness and falling asleep while driving, with some recounting experiences of near-misses and crashes: “*Well firsthand experience I’ve fallen asleep. I’ve actually had totalled my car driving home.*” (49). Many workers were also aware of the adverse effects of fatigue on their cognitive functioning and decision-making, which, in turn, affected their work performance. To compensate for this, they often reported adjusting their behavior by, for example, double-checking their own work and asking colleagues to review their decisions: “*I think I tend to ask others a little bit more readily, when I’m really tired. Like I don’t have quite the confidence in my decisions.*” (32). However, exceptions to this general pattern were reported in two studies (38, 41), as tunnelling workers and an emergency physician believed that fatigue did not negatively impact their work performance: “*And when I’m fatigued, I think I still make appropriate decisions and good medical management.*” (38).

Shift workers described how their fatigue was difficult to alleviate as work stressors functioned to perpetuate fatigue. Rumination about the past and worry about the future often made it difficult for sleep to happen naturally and automatically: “…*you’re just so tired, so worn out in your head, so everything that you just can’t manage yourself. You can’t even go to sleep.*” (37). This rumination and worry, frequently triggered by stressful events at work or upcoming shifts, suggested that shift workers found it hard to unwind and mentally detach from work: “*The only thing buzzing around in my head is did I do the right thing, did I give the right medication, things like that, and then I dream about these things.*” (31).

Many shift workers described feeling *“peer pressure to soldier through”* (28) shift work irrespective of how fatigued they were: “*You do what you have to do when you have to do it, regardless if you have slept or not.*” (33). Some workers reported that they wanted to avoid appearing “*weak*” (41), while nurses expressed how they wanted to be “*part of the team*” (51) and did not want to let colleagues down. Shift workers avoided disclosing their fatigue to their employer due to concerns about possible negative repercussions, such as potential disciplinary proceedings, underemployment, or being perceived as less capable: “…*you feel like you can’t tell them [employer] and it’s going to be a difficult thing and they might look at you and not give you shifts.*” (48). This perception of fatigue as normal, combined with the belief that admitting and discussing fatigue within the workplace shows weakness and a lack of camaraderie, appeared to perpetuate a detrimental workplace culture in which fatigue remains unacknowledged and untreated. As a result, many workers expressed the belief that fatigue is something they must silently accept and endure rather than to seek support for: “*I think it’s an expected thing and you know it’s going to happen to you and you just deal with it. No whining, no pain, no gain kind of thing. I think nurses just know it’s gonna happen and you’re pretty much just buck up and do it*.” (51).

### Balancing sleep needs with competing responsibilities

Shift workers described difficulties balancing daytime sleep with competing responsibilities, including family, work, and leisure: “*So I’m thinking, how am I going to juggle this? And I didn’t want to cancel stuff thinking… I could still fit it in, as insane as that is.*” (37). Family responsibilities, including childcare, were particularly disruptive to their sleep schedules. Although shift workers reported that they recognized the impact of insufficient sleep on their overall health and well-being (28), most said they chose to prioritize their families’ needs over their own sleep: “*Family comes first, and so my sleep would suffer over that because everything for the family is scheduled for the daytime.*” (33). However, the resulting fatigue from de-prioritizing sleep, and from shift work, often adversely impacted the quality of their time with family, leading to adverse emotional impacts such as “*being miserable, ‘snappy’ and having no energy*” (41). This appeared to cause strain on family relationships: “*…you go home and you’re not well-rested and you’re asked, ‘Where are we going to dinner tonight?’ and it seems like such an insignificant question, [but] that’s when a lot of people shut down and they just don’t talk and what’s important to your significant other you don’t care about.*” (54)

Shift workers reported difficulties getting adequate time for sleep between shifts, especially when work schedules did not allow enough time for sleep between shifts or regular sleep hours. They also reported difficulties getting adequate time for naps during shifts. High workloads were a commonly reported issue that contributed to *inconsistent, late or an absence of rest breaks* (45): “...*if you say ‘break’, it doesn’t exist”* (44). For those who managed to get breaks, they were often not able to nap in that short period of time to alleviate feelings of fatigue, while some were worried about sleep inertia after napping. Therefore, many chose to use breaks to relax or eat instead: “*Trying to eat and nap means you have to shovel in food and sleep longer or eat normally and sleep less. I can’t fall asleep that quickly so it’s hard for me to justify honking down food and facing reflux.*” (34).

Shift workers reported psychological and emotional consequences from trying to balance sleep with competing responsibilities. Those who tried to sleep during the day often felt guilty for not spending time with family, feeling as though they were *wasting their day sleeping* (33). They also worried about failing to meet important responsibilities while sleeping during the day and some would *keep their phone handy so that they could be reached and would answer any calls that came in* (34). Gender differences were evident, with women finding it particularly challenging to balance sleep, family, and work responsibilities because they face *gendered expectations about responsibility* (39): “*You have this mother-wife guilt that you’re sleeping all day*” (33).

Not all shift workers experienced these adverse impacts. Some workers believed that shift work offered greater flexibility to engage in activities such as exercising during the day, socializing with others who worked irregular hours, and spending more time with their children. This was generally reported by those who perceived they had more control over their work hours or those working part-time. Those with fewer competing responsibilities (eg, living alone or without children) often had more opportunities for daytime sleep: “*For me being single, I don’t have any kids. The most important thing is I sleep a lot when I’m at home.*” (54). For those with children, support from a partner or extended family was crucial for facilitating sleep by taking on more childcare responsibilities: “*My children knew I existed as a lump sleeping on the couch. Thank heaven for my husband who shouldered the parental load so well.*” (28)

### Obstacles to engaging in healthy behaviors

Shift workers reported that they knew which actions would reduce fatigue and benefit their sleep and health, but often found it difficult to translate this knowledge to their own behavior. For example, they were generally aware that excessive caffeine consumption, eating high calorie snacks, and not regularly engaging in physical activity were behaviors that only provided temporary relief from fatigue, and adversely impacted their sleep and longer-term health. Although they were seemingly aware of these adverse effects, shift workers often suggested they had limited control over preventing these unhealthy behaviors: “*I know…what’s healthy and what’s not. I can’t stop putting food into my mouth!! You know?...I know the types of exercise I could be doing to reduce it all, and knowing that doesn’t help and hasn’t helped, clearly.*” (53).

Shift workers frequently reported that food choices and intake were influenced by: (i) erratic work schedules that disrupted meal planning: “…*cooking becomes haphazard when working like this… I suppose it’s the irregular working hours*” (46), (ii) insufficient breaks at work, which led to on-the-go eating or skipping meals: “*Sometimes their [emergency service workers] only choice is to scoff a macca’s [McDonald’s] cheese burger or chocolate bar on the way to their next job”* (42), (iii) lack of healthy food options in the workplace, which increased reliance on more convenient fast-food options: “*I feel like sometimes we think, ‘Oh, I’m going to get something to eat,’ and then you eat and feel worse because of the [food] options that you have [at the hospital]*” (36), and (iv) inadequate facilities at work for storing home-cooked food: “*Previously I could make a salad or something like that to bring to work, but then I realized that we don’t have anywhere to store the food.*” (44).

Napping was another behavior that was perceived to be constrained by the shift work environment. While many workers recognized its benefits – such as *feeling energized or refreshed, improved mood, and clearer judgment* (32) – they often experienced obstacles, including inadequate facilities, insufficient breaks, or lack of managerial support: “*We have a problem with the inappropriate restroom in our ward. It has multiple uses as a restroom, a room for changing clothes, and a dining room. The coming and going of many people to it disturb our sleep and rest during the shift.*” (27)

Shift workers’ focus on external obstacles was reflected in their preferences for interventions which mainly focused on *organizational approaches to support fatigue management* (36), such as scheduled breaks or improved napping facilities, rather than individually-directed interventions. In two studies (48, 55), some shift workers were skeptical about behavioral interventions because they had found these interventions ineffective in the past or believed they were already engaging in healthy behaviors: *“She [a healthcare professional] gave me non-pharmacological approaches which of course wasn’t what I wanted […] I already did a lot of exercise and stuff. I wasn’t a coffee drinker or anything like that”* (55).

Few shift workers reported receiving sufficient education or training from their organization on how to manage fatigue and improve sleep. In the absence of formal education, workers relied on behavioral strategies they developed through personal experience over time. Some emphasized the importance of discovering strategies that work best for them: “*I think you just adapt to it and what is easiest for you. I didn’t read anything that said do 'this' or 'that'. It is just that the things that I do work best for me. It is just that after doing it for so many years and I have tried and tested different ways.*” (35)

Finally, shift workers reported that high levels of work-related stress and fatigue made it even harder to engage in healthy behaviors. For example, while some used exercise to manage stress and improve sleep, others felt too tired to exercise after shifts or on days off: “*If we had a busy night [in the station], then I’m saying scrap the gym, scrap golf, I’m going home, napping until noon, one, two, or whenever.*” (54). Despite efforts to be mindful of their diet, some workers frequently turned to unhealthy food choices as a way to cope with stress and fatigue: “*You have a bad day, and before you know it you have five (chocolates) in your mouth.*” (53). The combination of a demanding work environment, sleep disturbances, fatigue, and stress may compromise self-regulatory capacity, making it more difficult to engage in and sustain healthier behaviors.

### Confidence in the review findings

The three analytical themes were developed into short statements that described 22 review findings (table 4). Using the GRADE-CERQual approach, 10 review findings were assessed as high confidence, 11 as moderate confidence, and 1 as low confidence. We have provided an explanation for each GRADE-CERQual assessment in table 4 and full details are provided in the GRADE-CERQual Evidence Profile (supplementary appendix 6).

**Table 4:** GRADE-CERQual assessment of confidence (C) in the evidence – summary of qualitative findings (ordered from high-to-low C) within each theme. [HC=high confidence, MC=moderate confidence, LC=low confidence.]

Summary of review finding	Studies (reference) contributing to the review finding	GRADE-CERQual assessment	Explanation of GRADE-CERQual assessment
**Theme 1. Inevitability of fatigue and tiredness**			
Fatigue was perceived as an inevitable experience of shift work due to difficulties getting enough quality sleep when working non-standard hours and task-related fatigue associated with shift work.	N=19 (27–29, 31, 36–41, 43–45, 47–49, 51, 52, 54)	HC	19 studies with no or very minor concerns regarding methodological limitations, adequacy and relevance. There were minor concerns about coherence in two studies.
Shift workers recognized the consequences of fatigue on their physical and mental health.	N=14 (27–29, 31, 33, 36–39, 41, 43, 51, 52, 54)	HC	14 studies with no or very minor concerns regarding coherence, adequacy and relevance. There were minor concerns about methodological limitations in four studies.
Many shift workers were aware of the adverse effects of fatigue on their cognitive functioning and decision making, which, in turn, affected their work performance. To compensate for this, they often reported adjusting their behavior by, for example, double-checking their own work and asking colleagues to review their decisions. However, tunnelling workers and an emergency physician believed that fatigue did not negatively impact their work performance.	N=13 (27–29, 31, 32, 36–38, 40, 41, 50–52)	HC	13 studies with no or very minor concerns regarding coherence, adequacy and relevance. There were minor concerns about methodological limitations in four studies.
Shift workers experienced “peer pressure to soldier through” (37) fatigue and believed that they must silently accept and endure fatigue, rather than seek support for it.	N=14 (27–29, 33, 34, 36, 38, 40, 41, 43, 44, 48, 50, 51)	HC	14 studies with no or very minor concerns regarding coherence, adequacy and relevance. There were minor concerns about methodological limitations in six studies.
The perception of fatigue as normal, combined with the belief that admitting and discussing fatigue shows weakness and a lack of camaraderie, perpetuated a detrimental workplace culture in which fatigue remains unacknowledged and untreated. Shift workers avoided disclosing their fatigue to their employer due to concerns about possible negative repercussions.	N=12 (27–29, 33, 36, 40, 41, 44, 48, 50–52)	HC	12 studies with no or very minor concerns regarding methodological limitations, coherence and relevance. Although there were minor concerns regarding adequacy, these concerns did not significantly affect the review finding.
Shift workers often reported excessive sleepiness and falling asleep while driving, with some recounting experiences of near-misses and crashes.	N=11 (28, 30, 32–34, 40–42, 47, 49, 51)	MC	11 studies with no or very minor concerns regarding coherence and adequacy. However, there were moderate concerns regarding methodological limitations and relevance.
Shift workers struggled to unwind and mentally detach from work due to rumination and worry, frequently triggered by stressful work events or upcoming shifts, which often made it difficult for sleep to happen naturally and automatically.	N=11 (31, 34, 36–39, 41, 43, 45, 49, 52)	MC	11 studies with no or very minor concerns regarding coherence and adequacy. However, there were moderate concerns regarding methodological limitations and relevance.
**Theme 2. Balancing sleep needs with competing responsibilities**			
The resulting fatigue from de-prioritizing sleep, and from shift work, adversely impacted the quality of their time with family, leading to adverse emotional impacts and strain on family relationships.	N=14 (27, 28, 31, 33, 36–41, 43, 51, 52, 54)	HC	14 studies with no or very minor concerns regarding coherence, adequacy and relevance. There were minor concerns about methodological limitations in six studies.
Shift workers reported difficulties getting adequate time for sleep between shifts, especially when work schedules did not allow enough time for sleep between shifts.	N=19 (27, 31, 36, 38, 40, 41, 43, 45, 47, 48, 51, 52)	HC	19 studies with no or very minor concerns regarding methodological limitations, adequacy and relevance. There were minor concerns about coherence.
Shift workers described difficulties balancing daytime sleep with competing responsibilities, including family, work, and leisure. Family responsibilities, including childcare, were particularly disruptive to their sleep schedules. Although shift workers recognized the negative impact of insufficient sleep on their health and wellbeing, most prioritized their families’ needs over their own sleep. Shift workers with fewer competing responsibilities often had more opportunities for daytime sleep.	N=18 (27–29, 33, 36–41, 43, 45–49, 52, 54)	HC	18 studies with no or very minor concerns regarding adequacy and relevance. While there were minor concerns regarding methodological limitations and coherence, there were still a substantial number of strong and coherent studies that contributed to the review finding.
Shift workers reported difficulties getting adequate time for naps during shifts due to inconsistent, late, or an absence of rest breaks. When breaks were available, they were often too short to nap, while some would worry about sleep inertia after napping, leading shift workers to use breaks to relax or eat instead.	N=18 (29, 30, 32, 34–36, 38, 40–47, 49, 50, 53)	MC	18 studies with no or very minor concerns regarding coherence and adequacy. However, there were minor concerns regarding relevance, and moderate concerns regarding methodological limitations relating to eleven studies.
Shift workers reported psychological and emotional consequences from trying to balance sleep with competing responsibilities, such as guilt over missed family time and worrying about failing to meet important responsibilities while sleeping during the day.	N=12 (28, 31, 33–35, 37, 39, 43, 44, 50–52)	MC	12 studies with no or very minor concerns regarding coherence and adequacy. However, there were moderate concerns regarding methodological limitations and relevance.
Women found it particularly challenging to balance sleep and family responsibilities alongside their shift work due to gendered expectations.	N=4 (29, 33, 39, 54)	MC	4 studies with no or very minor concerns regarding coherence. However, there were moderate concerns regarding adequacy and minor concerns regarding methodological limitations and relevance.
Some shift workers felt that shift work provided greater flexibility in managing their time, especially those with more control over their hours or those working part-time.	N=9 (28, 31, 36, 37, 43, 46, 48, 50, 52)	MC	9 studies with no or very minor concerns regarding adequacy. However, there were moderate concerns regarding methodological limitations, and minor concerns regarding coherence and relevance.
For shift workers with children, support from a partner or extended family was crucial for facilitating sleep by taking on more childcare responsibilities.	N=7 (27–29, 33, 37–39)	MC	7 studies with no or very minor concerns regarding coherence. However, there were minor concerns regarding methodological limitations and relevance, and moderate concerns regarding adequacy.
**Theme 3: Obstacles to engaging in healthy behaviors**			
Shift workers preferred organizational interventions, such as scheduled breaks or improved napping facilities, over behavioral interventions. Some shift workers were skeptical about behavioral interventions because they had found them ineffective in the past or believed they were already engaging in healthy behaviors.	N=10 (27, 28, 31, 32, 36, 38, 47, 48, 50, 52)	HC	10 studies with no or very minor concerns regarding methodological limitations, adequacy and relevance. Although there were minor concerns about coherence, this was only due to a limited number of studies and there were no directly contradictory data.
Many shift workers recognized the benefits of napping at work but faced obstacles such as inadequate facilities, insufficient breaks, or lack of managerial support.	N=18 (27–36, 38, 40, 41, 44, 45, 47, 49, 50)	HC	18 studies with no or very minor concerns regarding coherence, adequacy and relevance. Although there were moderate concerns regarding methodological limitations due to ten studies, the review finding still is a valid representation of the data, in part due to the substantial number of studies contributing to this review finding.
Shift workers reported that high levels of work-related stress and fatigue made it even harder to engage in healthy behaviors. For example, while some shift workers used exercise to manage stress and improve sleep, others felt too tired to exercise after shifts or on days off. Despite efforts to be mindful of their diet, some workers frequently turned to unhealthy food choices as a way to cope with stress and fatigue.	N=9 (33, 35, 36, 38, 42, 44, 46, 53, 54)	HC	9 studies with no or very minor concerns regarding methodological limitations, coherence and adequacy. Although there were moderate concerns regarding relevance, the review finding is still a valid representation of the data.
Food choices and intake were influenced by erratic work schedules, insufficient breaks, lack of healthy food options, and inadequate facilities at work for storing home-cooked food.	N=12 (29, 35, 36, 38, 40, 42–47, 53)	MC	12 studies with no or very minor concerns regarding coherence and adequacy. However, there were minor concerns regarding relevance, and moderate concerns regarding methodological limitations.
Few shift workers reported receiving sufficient education or training from their organization on how to manage fatigue and improve sleep.	N=3 (47–49)	MC	3 studies with no or very minor concerns regarding methodological limitations and coherence, there were moderate concerns regarding adequacy and minor concerns regarding relevance.
Shift workers were aware of actions that would reduce fatigue and benefit their sleep and health, but often struggled to translate this knowledge into their own behavior.	N=14 (30, 31, 35, 36, 38, 40, 42–47, 49, 53)	MC	14 studies with no or very minor concerns regarding adequacy. However, there were minor concerns regarding methodological limitations and relevance, and moderate concerns regarding coherence.
Shift workers relied on behavioral strategies they developed through personal experience over time, emphasizing the importance of discovering strategies that work best for them.	N=3 (27, 33, 35)	LC	3 studies with no or very minor concerns regarding methodological limitations and coherence. However, there were moderate concerns regarding relevance and serious concerns regarding adequacy.

## Discussion

### Summary of the main findings

We developed three analytical themes from 28 studies on how shift workers experience sleep disturbance, fatigue and healthy behaviors. Shift workers perceive fatigue as inevitable and experience a workplace culture where they believe fatigue is something they must silently accept and endure rather than to seek support for. Shift workers struggle to balance their needs for daytime sleep with family and work responsibilities, often prioritizing family needs. Although shift workers know which actions would benefit their health and reduce fatigue, they often find it difficult to translate this knowledge into behavior.

### Comparison with the literature

Our findings suggest that a key challenge for shift workers in adopting behaviors that would reduce fatigue and benefit their health is a diminished capacity for self-regulation, due to fatiguing and stressful work environments. While previous research on shift workers has emphasized external factors like circadian misalignment, long working hours, and job demands, self-regulation issues have received less attention (42–44, 54). Previous studies have shown that insufficient sleep negatively affects self-regulation, with sleep-deprived individuals performing worse on self-regulation tasks involving working memory (56) and decision making (57). Our findings align with a recent review which proposed that being awake during the biological night increases impulsivity and negative affect, while weakening cognitive control, judgment, and emotional stability, thereby leading to cognitive and behavioral dysregulation (58).

This review has highlighted a workplace culture among shift workers of silently accepting fatigue as “part of the job”. Although this culture has been noted as a barrier to addressing fatigue among nurses (12, 59, 79) and the wider healthcare environment (60), little research has explored these cultural norms across different occupations. A recent online survey in Australia found that the majority of shift workers with a diagnosed sleep disorder adopted an “accept it and keep going” mentality as a fatigue management strategy (N=39, 97.5%) (61), but there is a lack of understanding of why shift workers are not actively seeking help. Our review suggests that the normalization of fatigue perpetuates a workplace culture where fatigue remains unacknowledged and untreated.

Our review found that family responsibilities play a crucial role in how shift workers manage sleep and fatigue. A recent study developed 18 healthy sleep practices for shift workers advised shift workers to “make sleep a priority by rescheduling social activities and household tasks where possible, and informing friends, family, and neighbors of your sleep schedule” [(62), p. 10]. However, our findings indicate that this may be unfeasible for many shift workers as they often prioritize family responsibilities above sleep to maintain strong relationships. Those who prioritized sleep sometimes experienced interpersonal and emotional challenges, such as strained relationships or difficulty conveying their need for daytime sleep to others. By contrast, supportive family environments helped them achieve adequate sleep.

### Strengths and limitations of the review

This is the first systematic qualitative evidence synthesis to examine shift workers’ experiences of sleep disturbance, fatigue and healthy behaviors across 20 different occupational groups (table 2). By synthesizing evidence from 28 studies, we captured a wide range of experiences, reducing the risk of any single study from overly influencing our understanding. A key strength was the involvement of a PPIEP group, which helped ensure the themes were relevant to shift workers’ lived experiences. Another methodological strength was our use of the GRADE-CERQual approach, which provided a transparent, standardized assessment of confidence in individual findings. This was particularly important given our inclusion of studies regardless of quality. While GRADE-CERQual evaluated the robustness of specific findings, our interpretative themes extended beyond descriptive data to offer new insights into shift workers’ experiences.

This review has some limitations. The inclusion of only English-language studies, which were predominantly from the UK, US and Australia, may underrepresent cultural differences. However, the review covered a broad range of occupations, shift types, and organizations, and thus speaks to a wide range of experiences. Only including peer-reviewed journal articles may increase the risk of dissemination biases (63). Finally, our review focused solely on the perspective of shift workers. Synthesizing qualitative research from the perspectives of shift workers’ family members would be particularly valuable, as they were identified as playing a key role in influencing shift workers’ sleep schedules.

### Implications for practice

Shift workers frequently cited organizational-level barriers to sleep and healthy behaviors. Therefore, organizational-level interventions are needed to create conditions in the workplace that support effective sleep and fatigue management, such as increased flexibility in work scheduling, quiet spaces for napping, and provision of healthier food options. Our review also highlights the importance of fostering a workplace culture that encourages open discussions about sleep-related issues. Changing the norm of silent acceptance is critical to address the range of sleep disorders among shift workers that often go unreported (64).

At the individual level, interventions that enhance behavioral, cognitive and emotional self-regulation could be promising. While self-regulatory techniques like goal-setting, self-monitoring, and feedback have shown positive effects on sleep quality in adults without clinical sleep disorders, they remain underutilized in sleep interventions (65). Since sleep loss and circadian disruption may influence brain functions related to self-regulation and propensity for negative repetitive thoughts (58), interventions addressing the interaction between individual vulnerabilities, sleep/circadian disruptions, and social-environmental factors may better mitigate shift work intolerance and sleep problems. Our findings, and existing evidence (66–70), suggest that education alone is insufficient. Sleep interventions that combine education with behavioral and psychological support have been shown to be more effective (70, 71).

Interventions for shift workers should address difficulties unwinding (pre-sleep cognitive arousal) and repetitive negative thinking about sleep, work, and responsibilities. Cognitive behavioral therapy for insomnia (CBT-I) may help manage maladaptive sleep beliefs, though its effectiveness for shift workers remains mixed (72). Acceptance and commitment therapy (ACT) offers an alternative approach by focusing on improving the relationship with negative thoughts rather than altering their content. This may be particularly suited to shift workers, since ACT promotes acceptance of work-related stressors while encouraging actions to reduce their impacts on thoughts and behaviors (73). Although ACT has shown promise in improving insomnia and sleep quality (74), evidence specific to shift workers remains limited.

Many existing sleep interventions overlook the role of family in supporting sleep schedules (75), which can undermine the acceptability and effectiveness of these interventions (66). To address this, interventions could include educational components aimed at helping families understand the importance of protecting sleep time, as recommended by UK National Institute for Health and Care Excellence (NICE) guidelines (76).

### Implications for future research

Only one study in this review explicitly targeted shift workers with a medically diagnosed sleep disorder (48). Shift work disorder (SWD) is a sleep disorder characterized by insomnia or excessive sleepiness and distress or impairment associated with shift work, lasting over three months (77). It affects approximately 27% of shift workers (78) and can exacerbate the negative effects and risks associated with shift work (79–82) and reduce quality of life (83). Despite its prevalence, none of the 28 studies included in this review screened participants for SWD. This gap in the literature limits our understanding of how experiences differ between shift workers with SWD and those who have either adapted more effectively or remain undiagnosed. Future research should explore these differences to determine how interventions can better support shift workers with SWD.

Finally, future research should explore the acceptability of interventions for shift workers, an area where there is a key need, but which remains under-investigated (84). Only two of the included studies in the present review focused on evaluating an intervention (34, 50). Given the skepticism towards non-pharmacological interventions identified in this review, co-designing interventions that address the specific needs of shift workers should be a priority. For example, while both CBT-I and ACT show promise for improving sleep outcomes, more research is needed to evaluate their acceptability for shift-working populations.

### Concluding remarks

This qualitative evidence synthesis found that shift workers perceive fatigue as inevitable and experience a workplace culture where fatigue remains unacknowledged, they struggle to balance daytime sleep with competing responsibilities, and they find it difficult to implement behaviors that would reduce fatigue and benefit their sleep and health. Behavioral sleep interventions should support shift workers to self-regulate their behavior, thoughts and emotions in fatiguing and stressful work environments, and provide behavioral and psychological support in addition to sleep education.

### Ethics approval

No ethical approval was needed since the systematic review is based on published data.

## Supplementary material

Supplementary material

## Data Availability

No new data were generated or analyzed in support of this research. NVivo file available upon request.
