# Supplementary material for: Shift workers’ experiences and views of sleep disturbance, fatigue and healthy behaviors: a systematic review and qualitative evidence synthesis
Source: Scand J Work Environ Health. 2025 Jun 26;51(4):282–97. doi: 10.5271/sjweh.4223 (PMC12281556; doi:10.5271/sjweh.4223)
Supplement: Supplementary material [file SJWEH-51-282-S001.pdf]

Shift workers' experiences and views of sleep disturbance, fatigue and healthy behaviors: a systematic review and qualitative evidence synthesis[1]

by Jack S Benton, PhD,[2] Charlotte L Lee, PhD, Hannah A Long, PhD, Thavapriya Sugavanam, PhD, Leah Holmes, PhD, Annie Keane, PhD, Neal Thurley, MA, Simon Kyle, PhD, David Ray, PhD, David P French, PhD

1. Supplementary material
2. Correspondence to: Jack Benton, Manchester Centre for Health Psychology, Division of Psychology & Mental Health, School of Health Sciences, Faculty of Biology, Medicine and Health, University of Manchester, Manchester, UK. [E-mail: jack.benton@manchester.ac.uk]

Appendix 1. PRISMA checklist

Section and Topic	Item #	Checklist item	Location where item is reported (page no.)
TITLE			
Title	1	Identify the report as a systematic review.	1,2,4
ABSTRACT			
Abstract	2	See the PRISMA 2020 for Abstracts checklist.	2
INTRODUCTION			
Rationale	3	Describe the rationale for the review in the context of existing knowledge.	3,4
Objectives	4	Provide an explicit statement of the objective(s) or question(s) the review addresses.	4
METHODS			
Eligibility criteria	5	Specify the inclusion and exclusion criteria for the review and how studies were grouped for the syntheses.	4-7
Information sources	6	Specify all databases, registers, websites, organisations, reference lists and other sources searched or consulted to identify studies. Specify the date when each source was last searched or consulted.	2,5, Appendix 3

Section and Topic	Item #	Checklist item	Location where item is reported (page no.)
Search strategy	7	Present the full search strategies for all databases, registers and websites, including any filters and limits used.	Appendix 3
Selection process	8	Specify the methods used to decide whether a study met the inclusion criteria of the review, including how many reviewers screened each record and each report retrieved, whether they worked independently, and if applicable, details of automation tools used in the process.	6
Data collection process	9	Specify the methods used to collect data from reports, including how many reviewers collected data from each report, whether they worked independently, any processes for obtaining or confirming data from study investigators, and if applicable, details of automation tools used in the process.	6
Data items	10a	List and define all outcomes for which data were sought. Specify whether all results that were compatible with each outcome domain in each study were sought (e.g. for all measures, time points, analyses), and if not, the methods used to decide which results to collect.	6
	10b	List and define all other variables for which data were sought (e.g. participant and intervention characteristics, funding sources). Describe any assumptions made about any missing or unclear information.	6
Study risk of bias assessment	11	Specify the methods used to assess risk of bias in the included studies, including details of the tool(s) used, how many reviewers assessed each study and whether they worked independently, and if applicable, details of automation tools used in the process.	6
Effect measures	12	Specify for each outcome the effect measure(s) (e.g. risk ratio, mean difference) used in the synthesis or presentation of results.	n/a
Synthesis methods	13a	Describe the processes used to decide which studies were eligible for each synthesis (e.g. tabulating the study intervention characteristics and comparing against the planned groups for each synthesis (item #5)).	7
	13b	Describe any methods required to prepare the data for presentation or synthesis, such as handling of missing summary statistics, or data conversions.	n/a
	13c	Describe any methods used to tabulate or visually display results of individual studies and syntheses.	n/a
	13d	Describe any methods used to synthesize results and provide a rationale for the choice(s). If meta-analysis was performed, describe the model(s), method(s) to identify the presence and extent of statistical heterogeneity, and software package(s) used.	7
	13e	Describe any methods used to explore possible causes of heterogeneity among study results (e.g. subgroup analysis, meta-regression).	n/a
	13f	Describe any sensitivity analyses conducted to assess robustness of the synthesized results.	n/a
Reporting bias assessment	14	Describe any methods used to assess risk of bias due to missing results in a synthesis (arising from reporting biases).	n/a

Section and Topic	Item #	Checklist item	Location where item is reported (page no.)
Certainty assessment	15	Describe any methods used to assess certainty (or confidence) in the body of evidence for an outcome.	7,8
RESULTS			
Study selection	16a	Describe the results of the search and selection process, from the number of records identified in the search to the number of studies included in the review, ideally using a flow diagram.	8,9, Figure 1
	16b	Cite studies that might appear to meet the inclusion criteria, but which were excluded, and explain why they were excluded.	8, Appendix 4
Study characteristics	17	Cite each included study and present its characteristics.	9, Table 2
Risk of bias in studies	18	Present assessments of risk of bias for each included study.	9, Table 3
Results of individual studies	19	For all outcomes, present, for each study: (a) summary statistics for each group (where appropriate) and (b) an effect estimate and its precision (e.g. confidence/credible interval), ideally using structured tables or plots.	n/a
Results of syntheses	20a	For each synthesis, briefly summarise the characteristics and risk of bias among contributing studies.	Table 4, Appendix 6
	20b	Present results of all statistical syntheses conducted. If meta-analysis was done, present for each the summary estimate and its precision (e.g. confidence/credible interval) and measures of statistical heterogeneity. If comparing groups, describe the direction of the effect.	10-16
	20c	Present results of all investigations of possible causes of heterogeneity among study results.	n/a
	20d	Present results of all sensitivity analyses conducted to assess the robustness of the synthesized results.	n/a
Reporting biases	21	Present assessments of risk of bias due to missing results (arising from reporting biases) for each synthesis assessed.	n/a
Certainty of evidence	22	Present assessments of certainty (or confidence) in the body of evidence for each outcome assessed.	Table 4, Appendix 6
DISCUSSION			

Section and Topic	Item #	Checklist item	Location where item is reported (page no.)
Discussion	23a	Provide a general interpretation of the results in the context of other evidence.	17,18
	23b	Discuss any limitations of the evidence included in the review.	18,19
	23c	Discuss any limitations of the review processes used.	18,19
	23d	Discuss implications of the results for practice, policy, and future research.	19-21
OTHER INFORMATION			
Registration and protocol	24a	Provide registration information for the review, including register name and registration number, or state that the review was not registered.	4
	24b	Indicate where the review protocol can be accessed, or state that a protocol was not prepared.	4
	24c	Describe and explain any amendments to information provided at registration or in the protocol.	n/a
Support	25	Describe sources of financial or non-financial support for the review, and the role of the funders or sponsors in the review.	22
Competing interests	26	Declare any competing interests of review authors.	22
Availability of data, code and other materials	27	Report which of the following are publicly available and where they can be found: template data collection forms; data extracted from included studies; data used for all analyses; analytic code; any other materials used in the review.	23

Appendix 2. ENTREQ checklist

No. Item	Guide questions/description	Reported on Page #
1. Aim	State the research question the synthesis addresses	2,4
2. Synthesis Methodology	Identify the synthesis methodology or theoretical framework which underpins the synthesis, and describe the rationale for choice of methodology (e.g. meta-ethnography, thematic synthesis, critical interpretive synthesis, grounded theory synthesis, realist synthesis, meta-aggregation, meta-study, framework synthesis)	7
3. Approach to strategy	Indicate whether the search was pre-planned (comprehensive search strategies to seek all available studies) or iterative (to seek all available concepts until they theoretical saturation is achieved)	5, Appendix 3
4. Inclusion criteria	Specify the inclusion/exclusion criteria (e.g. in terms of population, language, year limits, type of publication, study type)	4-5, Table 1
5. Data sources	Describe the information sources used (e.g. electronic databases (MEDLINE, EMBASE, CINAHL, PsycINFO), grey literature databases (digital thesis, policy reports), relevant organizational websites, experts, information specialists,	5, Appendix 3

	generic web searches (Google Scholar) hand searching, reference lists) and when the searches conducted; provide the rationale for using the data sources	
6. Electronic Search strategy	Describe the literature search (e.g. provide electronic search strategies with population terms, clinical or health topic terms, experiential or social phenomena related terms, filters for qualitative research, and search limits)	5, Appendix 3
7. Study screening methods	Describe the process of study screening and sifting (e.g. title, abstract and full text review, number of independent reviewers who screened studies)	6
8. Study Characteristics	Present the characteristics of the included studies (e.g. year of publication, country, population, number of participants, data collection, methodology, analysis, research questions)	9, Table 2
9. Study selection results	Identify the number of studies screened and provide reasons for study exclusion (e,g, for comprehensive searching, provide numbers of studies screened and reasons for exclusion indicated in a figure/flowchart; for iterative searching describe reasons for study exclusion and inclusion based on modifications to the research question and/or contribution to theory development)	9, Figure 1
10. Rationale for appraisal	Describe the rationale and approach used to appraise the included studies or	6

	selected findings (e.g. assessment of conduct (validity and robustness), assessment of reporting (transparency), assessment of content and utility of the findings)	
11. Appraisal items	State the tools, frameworks and criteria used to appraise the studies or selected findings (e.g. Existing tools: CASP, QARI, COREQ, Mays and Pope [25]; reviewer developed tools; describe the domains assessed: research team, study design, data analysis and interpretations, reporting)	6
12. Appraisal process	Indicate whether the appraisal was conducted independently by more than one reviewer and if consensus was required	6
13. Appraisal results	Present results of the quality assessment and indicate which articles, if any, were weighted/excluded based on the assessment and give the rationale	9, Table 3
14. Data extraction	Indicate which sections of the primary studies were analyzed and how were the data extracted from the primary studies? (e.g. all text under the headings “results /conclusions” were extracted electronically and entered into a computer software)	6
15. Software	State the computer software used, if any	7
16. Number of reviewers	Identify who was involved in coding and analysis	7
17. Coding	Describe the process for coding of data (e.g. line by line coding to search for	7

	concepts)	
18. Study comparison	Describe how were comparisons made within and across studies (e.g. subsequent studies were coded into pre-existing concepts, and new concepts were created when deemed necessary)	7
19. Derivation of Themes	Explain whether the process of deriving the themes or constructs was inductive or deductive	7
20. Quotations	Provide quotations from the primary studies to illustrate themes/constructs, and identify whether the quotations were participant quotations of the author's interpretation	10-16
21. Synthesis Output	Present rich, compelling and useful results that go beyond a summary of the primary studies (e.g. new interpretation, models of evidence, conceptual models, analytical framework, development of a new theory or construct)	10-16

Appendix 3. Search terms and search results by database

All databases were searched for inception until 01/08/2023. We used the Ovid interface to search MEDLINE (1946-present), EMBASE (1974-present) and PsycINFO (1806-present). We used the EBSCO interface to search CINAHL (1981-present). No date or language limits were applied. Sets of synonyms were produced in line with the distinct elements of our research question. We therefore selected thesaurus terms and free-text terms for each of the three aspects. Shift work is the first, followed by sleep and then either qualitative terms for methodologies or terms relating to experience. Synonyms for shift work included shift work schedule and work schedule tolerance. Synonyms for sleep included circadian and insomnia. Synonyms for qualitative methods included interview and grounded theory. Synonyms for experience included opinion and perspective. Full search strategies are provided below.

<u>CINAHL via EBSCO 1981 to 01/08/2023 – search strategy</u>

S1 (MH "Shiftwork") (4,570 hits)

S2 TI (((shift* or night*) n3 work*)) OR AB (((shift* or night*) n3 work*)) (6,120 hits)

S3 TI (((night* or rotat* or late or evening or irregular) n3 shift*)) OR AB (((night* or rotat* or late or evening or irregular) n3 shift*)) (3,122 hits)

S4 S1 OR S2 OR S3 (10,275 hits)

S5 (MH "Sleep+") (32,584 hits)

S6 (MH "Circadian Rhythm+") (7,787 hits)

S7 TI sleep* OR AB sleep* (80,386 hits)

S8 TI circadian OR AB circadian (5,631 hits)

S9 (MH "Sleep Disorders+") (45,337 hits)

S10 TI (insomnia* or dysomnia* or dyssomnia* or parasomnia* or hypersomnia*) OR AB (insomnia* or dysomnia* or dyssomnia* or parasomnia* or hypersomnia*) (12,152 hits)

S11 TI "night terror*" OR AB "night terror*" (62 hits)

S12 S5 OR S6 OR S7 OR S8 OR S9 OR S10 OR S11 (108,687 hits)

S13 MH "Qualitative Studies+" (182,499 hits)

S14 TI qualitative OR AB qualitative (171,837 hits)

S15 MH "Interviews+" (254,209 hits)

S16 TI interview* OR AB interview* (266,145 hits)

S17 TI "mixed method*" OR AB "mixed method*" (27,367 hits)

S18 MH "Thematic Analysis" (86,156 hits)

S19 TI thematic* OR AB thematic* (48,961 hits)

S20 TI theme* OR AB theme* (93,889 hits)

S21 TI "grounded theory" OR AB "grounded theory" (13,788 hits)

S22 MH "Anthropology, Cultural" (2,752 hits)

S23 TI ethnograph* OR AB ethnograph* (11,498 hits)

S24 MH "Content Analysis" (43,508 hits)

S25 TI "content analysis" OR AB "content analysis" (31,532 hits)

S26 MH "Discourse Analysis" (5,508 hits)

S27 TI "discourse analysis" OR AB "discourse analysis" (2,307 hits)

S28 (MH "Narratives") (20,535 hits)

S29 TI "narrative analysis" OR AB "narrative analysis" (1,526 hits)

S30 TI "conversation analysis" OR AB "conversation analysis" (760 hits)

S31 MH "Phenomenology" (4,214 hits)

S32 TI hermeneutic* OR AB hermeneutic* (5,121 hits)

S33 TI phenomenology OR AB phenomenology (4,982 hits)

S34 MH "Focus Groups" (50,613 hits)

S35 TI "focus group*" OR AB "focus group*" (45,148 hits)

S36 MH "Attitude+" (529,901 hits)

S37 TI attitude* OR AB attitude* (98,102 hits)

S38 TI view* OR AB view* (153,600 hits)

S39 MH "Life Experiences+" (57,508 hits)

S40 TI experienc* OR AB experienc* (554,178 hits)

S41 TI opinion* OR AB opinion* (42,611 hits)

S42 TI perce* OR AB perce* (459,767 hits)

S43 TI belie* OR AB belie* (106,872 hits)

S44 TI feel* OR AB feel* (70,717 hits)

S45 TI understand* OR AB understand* (353,886 hits)

S46 MH "Life Course Perspective" (227 hits)

S47 TI perspective* OR AB perspective* (170,977 hits)

S48 TI sentiment* OR AB sentiment* (3,088 hits)

S49 S13 OR S14 OR S15 OR S16 OR S17 OR S18 OR S19 OR S20 OR S21 OR S22 OR S23 OR S24 OR S25 OR S26 OR S27 OR S28 OR S29 OR S30 OR S31 OR S32 OR S33 OR S34 OR S35 OR S36 OR S37 OR S38 OR S39 OR S40 OR S41 OR S42 OR S43 OR S44 OR S45 OR S46 OR S47 OR S48 (1,946,558 hits)

S50 S4 AND S12 AND S49 (820 hits)

<u>Embase 1974 to 01/08/2023 – search strategy</u>

exp shift work/ (5,092 hits)

work schedule/ (10,096 hits)

((shift* or night*) adj3 work*).mp. (20,270 hits)

((night* or rotat* or late or evening or irregular) adj3 shift*).mp. (10,928 hits)

1 or 2 or 3 or 4 (32,389 hits)

exp sleep/ (289,941 hits)

circadian rhythm/ (98,976 hits)

sleep*.mp. (418,765 hits)

circadian.mp. (119,289 hits)

exp sleep disorder/ (301,374 hits)

(insomnia* or dysomnia* or dyssomnia* or parasomnia* or hypersomnia*).mp) (106,586 hits)

sleep deprivation/ (19,360 hits)

night terror*.mp. (430 hits)

6 or 7 or 8 or 9 or 10 or 11 or 12 or 13 (621,144 hits)

5 and 14 (11,191 hits)

exp qualitative research/ (117,136 hits)

qualitative analysis/ (84,165 hits)

qualitative.mp. (449,363 hits)

exp interview/ (366,952 hits)

interview*.mp. (633,863 hits)

mixed method*.mp. (48,299 hits)

thematic analysis/ (33,021 hits)

thematic*.mp. (88,456 hits)

theme*.mp. (174,130 hits)

grounded theory/ (10,125 hits)

grounded theory.mp. (19,264 hits)

cultural anthropology/ (55,581 hits)

ethnography/ (3,769 hits)

ethnograph*.mp. (16,351 hits)

content analysis/ (23,923 hits)

content analysis.mp. (52,954 hits)

discourse analysis/ (1,497 hits)

discourse analysis.mp. (3,532 hits)

narrative analysis.mp. (2,089 hits)

narrative/ (21,575 hits)

conversation analysis.mp. (1,083 hits)

hermeneutic*.mp. (5,332 hits)

phenomenology/ (13,332 hits)

phenomenology.mp. (21,657 hits)

focus group*.mp. (79,805 hits)

exp attitude/ (930,646 hits)

attitude*.mp. (605,441 hits)

view*.mp. (700,822 hits)

experience/ (41,152 hits)

experienc*.mp. (2,010,627 hits)

opinion*.mp. (185,273 hits)

perce*.mp. (2,047,823 hits)

belie*.mp. (456,615 hits)

feel*.mp. (178,915 hits)

understand*.mp. (1,922,587 hits)

life course perspective/ (321 hits)

perspective*.mp. (535,982 hits)

sentiment*.mp. (5,418 hits)

16 or 17 or 18 or 19 or 20 or 21 or 22 or 23 or 24 or 25 or 26 or 27 or 28 or 29 or 30 or 31 or 32 or 33 or 34 or 35 or 36 or 37 or 38 or 39 or 40 or 41 or 42 or 43 or 44 or 45 or 46 or 47 or 48 or 49 or 50 or 51 or 52 or 53 (7,805,113 hits)

15 and 54 (3,936 hits)

conference*.pt. (5,619,139 hits)

55 not 56 (2,823 hits)

Medline (Ovid MEDLINE® Epub Ahead of Print, In-Process & Other Non-Indexed Citations, Ovid MEDLINE® Daily and Ovid MEDLINE®) 1946 to 01/08/2023 – search strategy

Shift Work Schedule/ (1,038 hits)

Work Schedule Tolerance/ (7,536 hits)

((shift* or night*) adj3 work*).mp. (13,098 hits)

((night* or rotat* or late or evening or irregular) adj3 shift*).mp. (7,155 hits)

1 or 2 or 3 or 4 (20,720 hits)

exp Sleep/ (97,988 hits)

exp Circadian Rhythm/ (77,108 hits)

Circadian Clocks/ (5,243 hits)

sleep*.mp. (260,307 hits)

circadian.mp. (101,641 hits)

exp Sleep Wake Disorders/ (109,263 hits)

(insomnia* or dysomnia* or dyssomnia* or parasomnia* or hypersomnia*).mp. (33,706 hits)

night terror*.mp. (414 hits)

6 or 7 or 8 or 9 or 10 or 11 or 12 or 13 (357,376 hits)

5 and 14 (7,169 hits)

exp qualitative research/ (82,666 hits)

qualitative.mp. (345,548 hits)

interview/ (30,701 hits)

Interviews as Topic/ (66,831 hits)

interview*.mp. (490,626 hits)

mixed method*.mp. (42,234 hits)

thematic*.mp. (72,063 hits)

theme*.mp. (137,670 hits)

grounded theory/ (2,701 hits)

grounded theory.mp. (15,370 hits)

Anthropology, Cultural/ (7,462 hits)

ethnograph*.mp. (14,064 hits)

content analysis.mp. (43,477 hits)

discourse analysis.mp. (2,571 hits)

narrative analysis.mp. (1,819 hits)

personal narrative/ (6,212 hits)

Narration/ (10,175 hits)

conversation analysis.mp. (917 hits)

hermeneutics/ (569 hits)

hermeneutic*.mp. (4,746 hits)

phenomenology.mp. (11,723 hits)

Focus Groups/ (35,901 hits)

focus group*.mp. (70,072 hits)

exp Attitude/ (637,361 hits)

attitude*.mp. (483,434 hits)

view*.mp. (557,596 hits)

experienc*.mp. (1,420,089 hits)

opinion*.mp. (147,891 hits)

perce*.mp. (1,604,116 hits)

belie*.mp. (355,071 hits)

feel*.mp. (121,665 hits)

understand*.mp. (1,572,624 hits)

life course perspective/ (36 hits)

perspective*.mp. (453,217 hits)

sentiment*.mp. (5,486 hits)

16 or 17 or 18 or 19 or 20 or 21 or 22 or 23 or 24 or 25 or 26 or 27 or 28 or 29 or 30 or 31 or 32 or 33 or 34 or 35 or 36 or 37 or 38 or 39 or 40 or 41 or 42 or 43 or 44 or 45 or 46 or 47 or 48 or 49 or 50 (5,957,438 hits)

15 and 51 (2,351 hits)

<u>PsycINFO 1806 to 01/08/2023 – search strategy</u>

workday shifts/ (2,617 hits)

work scheduling/ (1,962 hits)

((shift* or night*) adj3 work*.mp) (6,067 hits)

((night* or rotat* or late or evening or irregular) adj3 shift*).mp) (2,084 hits)

1 or 2 or 3 or 4 (8,230 hits)

exp sleep/ (43,530 hits)

sleep wake cycle/ (4,935 hits)

sleep*.mp (100,538 hits)

circadian.mp (22,447 hits)

exp sleep wake disorders/ (24,139 hits)

(insomnia* or dysomnia* or dyssomnia* or parasomnia* or hypersomnia*).mp) (18,735 hits)

exp Sleep Aids/ (3,840 hits)

sleep deprivation/ (4,942 hits)

sleep talking/ (35 hits)

sleep terrors/ (1 hit)

sleep treatment/ (896 hits)

night terror*.mp) (357 hits)

6 or 7 or 8 or 9 or 10 or 11 or 12 or 13 or 14 or 15 or 16 or 17 (1,275,279 hits)

exp qualitative methods/ (21,097 hits)

qualitative measures/ (111 hits)

qualitative.mp (225,883 hits)

exp interviews/ (19,474 hits)

interview*.mp (485,872 hits)

mixed methods research/ (831 hits)

mixed method*.mp (35,854 hits)

thematic*.mp (49,950 hits)

theme*.mp (148,127 hits)

grounded theory.mp (19,606 hits)

ethnography/ (11,138 hits)

ethnograph*.mp (34,577 hits)

exp content analysis/ (22,326 hits)

content analysis.mp (34,976 hits)

discourse analysis.mp (14,703 hits)

narrative analysis.mp (3,472 hits)

narratives/ (25,290 hits)

conversation analysis.mp (2,828 hits)

hermeneutics/ (2,489 hits)

hermeneutic*.mp (8,731 hits)

phenomenology/ (18,199 hits)

phenomenology.mp (28,541 hits)

focus group*.mp (47,620 hits)

exp attitudes/ (444,930 hits)

attitude*.mp (544,882 hits)

view*.mp (344,752 hits)

exp "experiences (events)"/ (176,283 hits)

experienc*.mp (842,773 hits)

opinion*.mp (64,677 hits)

perce*.mp (940,154 hits)

belie*.mp (248,794 hits)

feel*.mp (172,173 hits)

understand*.mp (640,085 hits)

perspective*.mp (338,443 hits)

sentiment*.mp (9,426 hits)

19 or 20 or 21 or 22 or 23 or 24 or 25 or 26 or 27 or 28 or 29 or 30 or 31 or 32 or 33 or 34 or 35 or 36 or 37 or 38 or 39 or 40 or 41 or 42 or 43 or 44 or 45 or 46 or 47 or 48 or 49 or 50 or 51 or 52 or 53 (2,971,259 hits)

5 and 18 and 54 (1,184 hits)

Appendix 4. Articles excluded at the full-text screening stage

Exclusion reason: Not English

1. Caixete, C., Borges, C. R. C., Iwamoto, H. H., & Carmago, F. C. (2012). Is there fatigue caused by night shift working among nursing professionals? *Saude Coletiva, 9*(57), 89–93.

2. Furon, D., Frimat, P., & Caillard, J. F. (1978). Medical, social, and professional aspects of shift working in women. *Archives des Maladies Professionnelles de Médecine du Travail et de Sécurité Sociale, 39*(3), 121–139.

3. Revista Paulista de Enfermagem. (2009). Influence of the work shift in daily life of nursing professional women. *Revista Paulista de Enfermagem*, 28(1), 1p–1p.

Exclusion reason: Not peer-reviewed

4. Long shifts, lack of sleep fatigue health care workers: Changing attitudes about rest and reasonable work schedules. (2011). *Joint Commission Perspectives on Patient Safety*, 11(3), 9–11.

5. McMillan, D. E., & Fallis, W. M. (2011). Benefits of napping on night shifts. *Nursing Times, 107*(44), 12-13.

6. Sargent, S. A. (2013). Asleep behind the wheel: Experiences of night shift nurses on the commute home (Ph.D. dissertation).

Exclusion reason: No substantive qualitative element

7. Adams, J., Folkard, S., & Young, M. (1986). Coping strategies used by nurses on night duty. *Ergonomics, 29*(2), 185–196.

8. Alsayed, S. A., Abou Hashish, E. A., & Alshammari, F. (2022). Occupational fatigue and associated factors among Saudi nurses working 8-hour shifts at public hospitals. *SAGE Open Nursing, 8*.

9. Books, C., Coody, L. C., Kauffman, R., & Abraham, S. (2020). Night shift work and its health effects on nurses. *The Health Care Manager, 39*(3), 122–127.

10. Carney, M. L. (2015). How the other half lives. *Nursing Management, 46*(7), 30–35.

11. Clissold, G., & Smith, P. (2002). A study of female nurses combining partner and parent roles with working a continuous three-shift roster: The impact on sleep, fatigue, and stress. *Contemporary Nurse, 12*(3), 294–302.

12. Cranley, N. M., Cunningham, C. J. L., & Panda, M. (2016). Understanding time use, stress, and recovery practices among early career physicians: An exploratory study. *Psychology, Health & Medicine, 21*(3), 362–367.

13. Dore, E., Guerero, D., Wallbridge, T., Holden, A., Anwar, M., Eastaugh, A., Desai, D., & Clare, S. (2021). Sleep is the best medicine: How rest facilities and EnergyPods can improve staff wellbeing. *Future Healthcare Journal, 8*(3), e625–e628.

14. Farag, A., Scott, L. D., Perkhounkova, Y., Saeidzadeh, S., & Hein, M. (2022). A human factors approach to evaluate predictors of acute care nurse occupational fatigue. *Applied Ergonomics, 100,* 103647.

15. Garde, A. H., Nabe-Nielsen, K., & Aust, B. (2011). Influence on working hours among shift workers and effects on sleep quality: An intervention study. *Applied Ergonomics, 42*(2), 238–243.

16. Geiger-Brown, J., Sagherian, K., Shijun, Z., Wieroniey, M. A., Blair, L., Warren, J., Hinds, P. S., & Szeles, R. (2016). Napping on the night shift: A two-hospital implementation project. *AJN American Journal of Nursing, 116*(5), 26–34.

17. Harrison, E. M., Schmied, E. A., Easterling, A. P., Yablonsky, A. M., & Glickman, G. L. (2020). A hybrid effectiveness-implementation study of a multi-component lighting intervention for hospital shift workers. *International Journal of Environmental Research and Public Health, 17*(23).

18. Khaleque, A., & Rahman, A. (1982). Sleep disturbances and health complaints of shift workers. *Journal of Human Ergology, 11*(Suppl), 155-164.

19. Koller, M., Kundi, M., & Cervinka, R. (1978). Field studies of shift work at an Austrian oil refinery. I. Health and psychosocial wellbeing of workers who drop out of shiftwork. *Ergonomics, 21*(10), 835-847.

20. Loew, M., Niel, K., Burlison, J. D., Russell, K. M., Karol, S. E., Talleur, A. C., Christy, L. A. N. N., Johnson, L.-M., & Crabtree, V. M. (2019). A quality improvement project to improve pediatric medical provider sleep and communication during night shifts. *International Journal for Quality in Health Care, 31*(8), 633-638.

21. Maasen, A., Meers, A., & Verhaegen, P. (1980). Quantitative and qualitative aspects of sleep in young self-selected four-shift workers. *International Archives of Occupational and Environmental Health, 45*(1), 81-86.

22. Meulenbergs, M., & Verhaegen, P. (1982). Quantitative and qualitative aspects of sleep in 50-56 years old self-selected shiftworkers. *Journal of Human Ergology, 11*(Suppl), 457-464.

23. Riley, K., Nazareno, J., & Malish, S. (2016). 24-hour care: Work and sleep conditions of migrant Filipino live-in caregivers in Los Angeles. *American Journal of Industrial Medicine, 59*(12), 1120-1129.

24. Soares, M. M., Jacobs, K., Rathore, H., Shukla, K., Singh, S., & Tiwari, G. (2012). Shift work: Problems and its impact on female nurses in Udaipur, Rajasthan, India. *Work, 41*, 4302-4314.

25. Zaslona, J. L., O'Keeffe, K. M., Signal, T. L., & Gander, P. H. (2018). Shared responsibility for managing fatigue: Hearing the pilots. *PLOS ONE, 13*(5), e0195530.

Exclusion reason: Not related to sleep

26. Argyriadis, A., Ioannidou, L., Dimitrakopoulos, I., Gourni, M., Ntimeri, G., Vlachou, C., & Argyriadi, A. (2023). Experimental mindfulness intervention in an emergency department for stress management and development of a positive working environment. *Healthcare, 11*(6).

27. Brown, M., Tucker, P., Rapport, F., Hutchings, H., Dahlgren, A., Davies, G., & Ebden, P. (2010). The impact of shift patterns on junior doctors' perceptions of fatigue, training, work/life balance, and the role of social support. *Quality & Safety in Health Care, 19*(6), e36.

28. Dall'Ora, C., Sainsbury, J., & Allen, C. (2022). Student nurses' views on shift patterns: What do they prefer and why? Results from a Tweetchat. *Nursing Open, 9*(3), 1785–1793.

29. Greenfield, R., Busink, E., Wong, C. P., Riboli-Sasco, E., Greenfield, G., Majeed, A., Car, J., & Wark, P. A. (2016). Truck drivers' perceptions on wearable devices and health promotion: A qualitative study. *BMC Public Health, 16,* 677.

30. Ihara, Y., Son, D., Nochi, M., & Takizawa, R. (2020). Work-related stressors among hospital physicians: A qualitative interview study in the Tokyo metropolitan area. *BMJ Open, 10*(9), e034848.

31. Mathew, R., Gundy, S., Ulic, D., Haider, S., & Wasi, P. (2016). A reduced duty hours model for senior internal medicine residents: A qualitative analysis of residents' experiences and perceptions. *Academic Medicine, 91*(9), 1284-1292.

32. Maynard, S., Miller, K. A., & Filtness, A. (2022). Keeping the service running: Examining working relationships and workload of London bus network iBus controllers. *Applied Ergonomics, 98*, 103577.

33. McGowan, Y., Humphries, N., Burke, H., Conry, M., & Morgan, K. (2013). Through doctors' eyes: A qualitative study of hospital doctor perspectives on their working conditions. *British Journal of Health Psychology, 18*(4), 874-891.

34. Naweed, A., Chapman, J., Allan, M., & Trigg, J. (2017). It comes with the job: Work organizational, job design, and self-regulatory barriers to improving the health status of train drivers. *Journal of Occupational and Environmental Medicine, 59*(3), 264-273.

35. Novak, R. D., & Auvil-Novak, S. E. (1996). Focus group evaluation of night nurse shiftwork difficulties and coping strategies. *Chronobiology International, 13*(6), 457-463.

36. Oftedal, S., Burrows, T., Fenton, S., Murawski, B., Rayward, A. B., & Duncan, M. J. (2019). Feasibility and preliminary efficacy of an m-Health intervention targeting physical activity, diet, and sleep quality in shift-workers. *International Journal of Environmental Research and Public Health, 16*(20).

37. Ogeil, R. P., Savic, M., Ferguson, N., & Lubman, D. I. (2021). Shift-work-play: Understanding the positive and negative experiences of male and female shift workers to inform opportunities for intervention to improve health and wellbeing. *Australian Journal of Advanced Nursing, 38*(2), 23-33.

38. Ram, A., Kurpad, A., & Swaminathan, S. (2014). Understanding the health behaviors of India's information technology and business process outsourcing employees. *International Journal of Workplace Health Management, 7*(4), 213-228.

39. Savic, M., Ogeil, R. P., Sechtig, M. J., Lee-Tobin, P., Ferguson, N., & Lubman, D. I. (2019). How do nurses cope with shift work? A qualitative analysis of open-ended responses from a survey of nurses. *International Journal of Environmental Research and Public Health, 16*(20).

40. Stimpfel, A. W., Liang, E., & Goldsamt, L. A. (2020). Early career nurse reports of work-related substance use. *Journal of Nursing Regulation, 11*(1), 29-35.

41. Sugimoto, M., & Bayrampour, H. (2022). Experience of pregnancy during family medicine residency: A qualitative research study. *Canadian Family Physician, 68*(5), 356-363.

42. Suleiman, A. O., Decker, R. E., Garza, J. L., Laguerre, R. A., Dugan, A. G., & Cavallari, J. M. (2021). Worker perspectives on the impact of non-standard workdays on worker and family well-being: A qualitative study. *BMC Public Health, 21*(1), 2230.

43. Suresh, N., & Srinivas, V. (2020). Exploring the dream pattern among the nightshift workers: A qualitative study. *International Journal of Dream Research, 13*(2), 143-150.

44. Taylor, J., Stratton, E., McLean, L., Richards, B., & Glozier, N. (2022). How junior doctors perceive personalised yoga and group exercise in the management of occupational and traumatic stressors. *Postgraduate Medical Journal, 98*(1161), e10.

45. Thwaite, T. L., Heidke, P., Williams, S. L., Vandelanotte, C., Rebar, A. L., & Khalesi, S. (2020). Barriers to healthy lifestyle behaviors in Australian nursing students: A qualitative study. *Nursing & Health Sciences, 22*(4), 921-928.

46. Torkington, A. M., Larkins, S., & Gupta, T. S. (2011). The psychosocial impacts of fly-in fly-out and drive-in drive-out mining on mining employees: A qualitative study. *Australian Journal of Rural Health, 19*(3), 135-141

47. Wenger, J. (2008). Freedom isn't free: Voices from the truck driving industry. *New Solutions, 18*(4), 481-491.

48. West, S., Boughton, M., & Byrnes, M. (2009). Juggling multiple temporalities: The shift work story of mid-life nurses. *Journal of Nursing Management, 17*(1), 110-119.

49. West, S., Rudge, T., & Mapedzahama, V. (2021). Temporalities of night shift work: The power of sleep for midlife nurses. *Nursing Inquiry, 28*(2), e12410.

Exclusion reason: Not shift worker population

50. Coveney, C. M. (2014). Managing sleep and wakefulness in a 24-hour world. *Sociology of Health & Illness, 36*(1), 123–136.

51. Crowley, S. K., Wilkinson, L. L., Burroughs, E. L., Muraca, S. T., Wigfall, L. T., Louis-Nance, T., Williams, E. M., Glover, S. H., & Youngstedt, S. D. (2012). Sleep during basic combat training: A qualitative study. *Military Medicine, 177*(7), 823–828.

52. Goheer, A., Holzhauer, K., Martinez, J., Woolf, T., Coughlin, J. W., Martin, L., Zhao, D., Lehmann, H., Clark, J. M., & Bennett, W. L. (2021). What influences the “when” of eating and sleeping? A qualitative interview study. *Appetite, 156,* 104980.

53. Mojtahedzadeh, N., Rohwer, E., Neumann, F. A., Nienhaus, A., Augustin, M., Zyriax, B.-C., Harth, V., & Mache, S. (2021). The health behaviour of German outpatient caregivers in relation to their working conditions: A qualitative study. *International Journal of Environmental Research and Public Health, 18*(11).

54. Shan, D., & Neis, B. (2020). Employment-related mobility, regulatory weakness and potential fatigue-related safety concerns in short-sea seafaring on Canada's Great Lakes and St. Lawrence Seaway: Canadian seafarers' experiences. *Safety Science, 121*, 165-176.

55. Smith, L., & Folkard, S. (1993). The perceptions and feelings of shiftworkers' partners. *Ergonomics, 36*(1-3), 299-305.

56. Van Den Berg, M. J., Signal, T. L., & Gander, P. H. (2020). Fatigue risk management for cabin crew: The importance of company support and sufficient rest for work-life balance—a qualitative study. *Industrial Health, 58*(1), 2-14.

57. Wang, Z., Li, M. K., Zhang, Q., Wang, Y., & Zhang, W. (2023). High-speed train drivers' operation performance: Key factors, models, and management implications. *International Journal of Industrial Ergonomics, 97*, 103482.

Appendix 5. Thematic map of findings

Analytical themes		
Inevitability of fatigue and tiredness	Balancing sleep needs with competing responsibilities	Obstacles to engaging in healthy behaviors

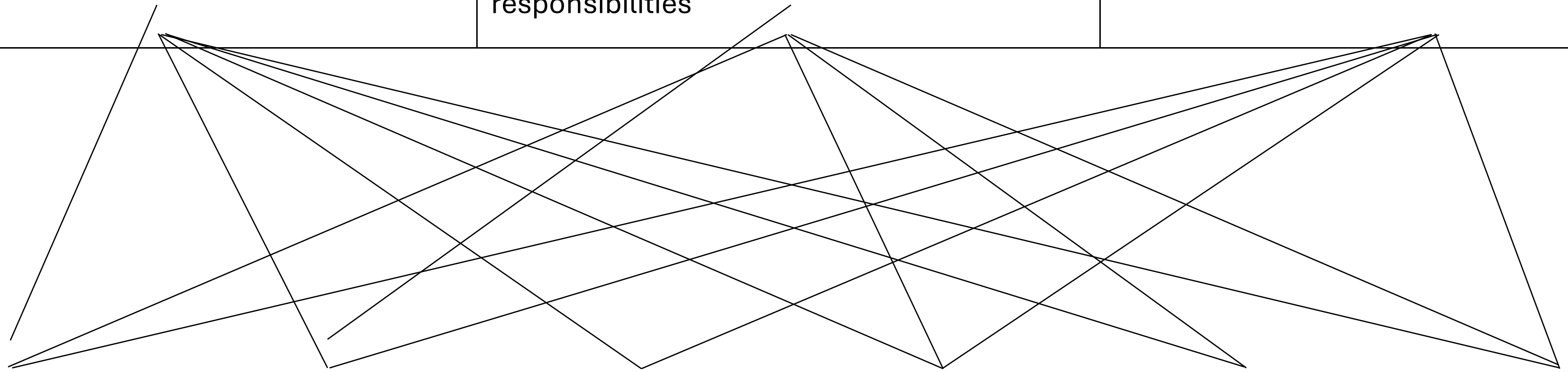

Descriptive themes					
Shift work is the cause of sleep disturbance and fatigue	Negative impacts of shift work, sleep disturbance and fatigue	Cultural norms and practices in the workplace	Juggling sleep, shift work and other responsibilities	Psychological and emotional challenges	Strategies for managing sleep and fatigue
Codes					

Attributing sleep issues to shift work (1st, 2nd)<br><br>Being on call impacts sleep (1st, 2nd)<br><br>Challenges adapting sleep to different time zones (1st)<br><br>Eating habits influenced by shift work (1st, 2nd)<br><br>Impact of commuting on fatigue (1st, 2nd)<br><br>Impact of shift work on sleep and fatigue (1st, 2nd)<br><br>Inadequate breaks affecting dietary behavior (1st, 2nd)<br><br>Inadequate food facilities at work (1st, 2nd)<br><br>Inadequate napping facilities at work (1st, 2nd)<br><br>Inadequate rest areas at work (1st, 2nd)<br><br>Inconsistent, interrupted, or lack of breaks (1st, 2nd)	Always feeling switched on at work (1st, 2nd)<br><br>Difficulties sleeping before shifts (1st)<br><br>Driving while drowsy (1st, 2nd)<br><br>Fatigue does not impact work performance (1st, 2nd)<br><br>Fatigue from social interactions at work (2nd)<br><br>Fatigue influences dietary choices (1st, 2nd)<br><br>Feeling too tired to exercise (1st, 2nd)<br><br>Health impacts of shift work and sleep disturbance (1st, 2nd)<br><br>Impact of fatigue on career choices (1st, 2nd)<br><br>Impact of fatigue on compassion and patient care (1st, 2nd)<br><br>Impact of fatigue on work performance and errors (1st, 2nd)	Colleagues support with workload when fatigued (1st, 2nd)<br><br>Concerns about patient care when napping at work (1st, 2nd)<br><br>Cultural norms in the workplace impacting breaks (1st, 2nd)<br><br>Fear of employer reaction to seeking help for sleep and fatigue issues (1st, 2nd)<br><br>Feeling responsible for other people (1st, 2nd)<br><br>Lack of communication and support among colleagues regarding fatigue (1st, 2nd)<br><br>Napping is acceptable at work (1st, 2nd)<br><br>Napping is not allowed at work (2nd)<br><br>Not seeking help for sleep issues (2nd)<br><br>Perceived stigma with admitting fatigue (1st, 2nd)	Being too tired to spend time with family and friends (1st, 2nd)<br><br>Challenges incorporating physical activity around shift work and other responsibilities (1st, 2nd)<br><br>Childcare duties contributing to fatigue (1st, 2nd)<br><br>Desire to help partner with household responsibilities (1st, 2nd)<br><br>Difficulties balancing sleep, family, and work responsibilities (1st, 2nd)<br><br>Expectations for family responsibilities (1st, 2nd)<br><br>Gender differences in family responsibilities (2nd)<br><br>Home environment not conducive to daytime sleeping (1st, 2nd)<br><br>Importance of a supportive partner for facilitating sleep (1st, 2nd)	Difficulties unwinding from work (1st, 2nd)<br><br>Feeling guilty about sleeping at work (1st, 2nd)<br><br>Feeling guilty about sleeping during the day (1st, 2nd)<br><br>Feeling guilty about unfulfilled family responsibilities (1st, 2nd)<br><br>Feeling guilty for leaving work with colleagues (1st, 2nd)<br><br>Feeling isolated in managing fatigue (1st, 2nd)<br><br>Importance of family and friends for emotional support (1st, 2nd)<br><br>Ruminating about work (1st, 2nd)<br><br>Social isolation due to shift work (1st, 2nd)<br><br>Strains on relationships due to fatigue and shift work (1st, 2nd)	Acknowledging the importance of taking a break (1st, 2nd)<br><br>Adapting sleep patterns to manage shift work fatigue (1st, 2nd)<br><br>Adapting to shift work fatigue over time (1st, 2nd)<br><br>Alcohol use for sleep and relaxation (1st, 2nd)<br><br>Avoiding alcohol to prevent exacerbating fatigue (2nd)<br><br>Behavioral interventions perceived as ineffective (1st, 2nd)<br><br>Benefits of physical activity for sleep and fatigue management (1st, 2nd)<br><br>Delayed sleep onset when trying to nap (2nd)<br><br>Desire for support in accessing sleep treatment (2nd)

Insufficient sleep between shifts (1st, 2nd)<br><br>Irregular work schedule = irregular eating patterns (1st, 2nd)<br><br>Lack of time for food preparation outside of shift work (1st, 2nd)<br><br>Lack of time to eat at work (1st, 2nd)<br><br>Lack of time to nap at work (1st, 2nd)<br><br>Lack of time to unwind at home (1st, 2nd)<br><br>Lack of organisational and managerial support for sleep (1st, 2nd)<br><br>Limited access to healthy food in the workplace (1st, 2nd)<br><br>Negative impact of shift schedules (1st, 2nd)<br><br>Opportunities for physical activity due to shift work (1st, 2nd)<br><br>Perceived inevitability of sleep disturbance and	Incidents of drowsy driving (1st, 2nd)<br><br>Increased caution at work when fatigued (1st, 2nd)<br><br>Negative emotional impacts of shift work, fatigue and sleep issues (1st, 2nd)<br><br>Negative impact of light (1st, 2nd)<br><br>Negative impacts of unhealthy eating habits (1st, 2nd)<br><br>Post-work exhaustion (1st, 2nd)<br><br>Stressful meal times at work (1st, 2nd)	Social pressures against napping at work (2nd)<br><br>Soldiering through shift work regardless of fatigue (1st, 2nd)<br><br>Women feel unsafe when napping (1st, 2nd)	Missing family and social events due to shift work (1st, 2nd)<br><br>Planning dedicated time with family (1st, 2nd)<br><br>Prioritization of physical activity vs. sleep (1st, 2nd)<br><br>Sacrificing sleep for family and social events (1st, 2nd)<br><br>Shift work helps meet family responsibilities (1st, 2nd)<br><br>Sleep being interrupted by family and friends (1st, 2nd)	Worrying about family and household responsibilities (2nd)<br><br>Worrying about limited time to sleep (1st, 2nd)<br><br>Worrying about shift work (1st, 2nd)	Difficulties accessing help for sleep issues (1st, 2nd)<br><br>Eating to manage fatigue and stress (1st, 2nd)<br><br>Excessive alcohol consumption contributing to fatigue (1st, 2nd)<br><br>Food planning and preparation facilitating healthy eating at work (1st, 2nd)<br><br>Hesitancy about using caffeine to manage fatigue (1st, 2nd)<br><br>Importance of a trusted and understanding HCP when seeking help (1st, 2nd)<br><br>Importance of understanding what works best for them (1st, 2nd)<br><br>Ineffective strategies to combat drowsy driving (1st, 2nd)<br><br>Keeping busy and active to manage fatigue (1st, 2nd)

fatigue due to shift work (1st, 2nd)<br><br>Positive impacts of shift schedule modification (1st, 2nd)<br><br>Shift schedules preventing access to seeking help from a healthcare professional (1st, 2nd)<br><br>Staffing issues contribute to fatigue (1st, 2nd)					Lack of education on sleep and fatigue management (1st, 2nd)<br><br>Napping to manage fatigue (1st, 2nd)<br><br>Negative experience of seeking help for sleep issues (1st, 2nd)<br><br>Perceived benefits of napping at work (1st, 2nd)<br><br>Positive experience of seeking help for sleep issues (1st, 2nd)<br><br>Preparing the home environment to facilitate sleep (1st, 2nd)<br><br>Self-care strategies to manage fatigue (1st, 2nd)<br><br>Sleep inertia after napping (1st, 2nd)<br><br>Strategies to stay alert while driving (1st, 2nd)<br><br>Trying to keep a regular sleep pattern (2nd)<br><br>Using caffeine to manage fatigue at work (1st, 2nd)

					Using physical activity to manage stress (1st, 2nd)<br><br>Using the commute for physical activity (1st, 2nd)<br><br>Winding down to improve sleep (1st, 2nd)

1st: First-order code

2nd: Second-order code

Appendix 6. GRADE-CERQual Evidence profile

Summary of review finding	Studies (reference) contributing to the review finding	Methodological limitations	Coherence	Adequacy	Relevance	Confidence in the evidence	Explanation of GRADE-CERQual assessment
Theme 1. Inevitability of fatigue							
Fatigue was perceived as an inevitable experience of shift work due to difficulties getting enough quality sleep when working non-standard hours and task-related fatigue associated with shift work.	n=19<br><br>(28-30, 32, 37-42, 44-46, 48-50, 52, 53, 55)	No or very minor concerns<br><br>2 studies had limitations in the study design, recruitment strategy, and data collection methods. 4 studies did not conduct a sufficiently rigorous analysis. Nonetheless, there were only very minor concerns because these limitations applied to only 5 of 19 studies and therefore did not	Minor concerns<br><br>The majority of data (from 17 studies) directly supported this review finding. 1 study suggested that a diagnosis of sleep disorder can also contribute to fatigue. In another study, participants described how they limited consecutive shifts to cope with fatigue, a perspective that could potentially challenge the	No or very minor concerns<br><br>19 studies contributed to this review finding and represented a variety of examples that illustrated or explained the finding. Although a minority of data contradicted the review finding (see coherence), the other findings still provided rich underlying data.	No or very minor concerns<br><br>13 of the 19 studies that contributed to the review finding were directly relevant to the review question on shift workers' experiences of sleep disturbance and strategies for managing fatigue, while 5 studies were indirectly relevant as they focused on broader lifestyle and work-related	High confidence	19 studies contributed to the review finding, with no or very minor concerns regarding methodological limitations, adequacy and relevance. Although there were minor concerns about coherence, this was only due to 2 studies.

Summary of review finding	Studies (reference) contributing to the review finding	Methodological limitations	Coherence	Adequacy	Relevance	Confidence in the evidence	Explanation of GRADE-CERQual assessment
		affect the review finding notably.	notion of fatigue as an "inevitable" consequence rather than a manageable challenge. However, there were no contradictory data in the other 17 studies.		factors. 1 study was partially relevant as it focused on dietary behaviors. The studies include a diverse range of occupations (including nurses, midwives, paramedics, emergency physicians, emergency service personnel, night-time economy workers, bus drivers, firefighters, flight attendants, and tunnelling workers). There were a range of shift types, although 5 studies did not report shift type. 13 of the 19 studies were from		

Summary of review finding	Studies (reference) contributing to the review finding	Methodological limitations	Coherence	Adequacy	Relevance	Confidence in the evidence	Explanation of GRADE-CERQual assessment
					the UK or US, although there were 7 different countries in total. Therefore, we had no or very minor concerns about relevance.		
Shift workers recognized the consequences of fatigue on their physical and mental health.	n=14 (28-30, 32, 34, 37-40, 42, 44, 52, 53, 55)	Minor concerns<br><br>1 study had limitations in its study design, recruitment strategy, and data collection methods. 4 studies did not conduct a sufficiently rigorous analysis. We had minor concerns because these limitations applied to 4 of 14 studies.	No or very minor concerns<br><br>The underlying data directly and unambiguously supported the review finding and there were no contradictory data.	No or very minor concerns<br><br>14 studies contributed to this review finding and represented a variety of examples that illustrated or explained the finding from a diverse range of both physical and mental health impacts.	No or very minor concerns<br><br>10 of the 14 studies that contributed to the review finding were directly relevant to the review question on shift workers' experiences of sleep disturbance and strategies for managing fatigue, while 4 studies were indirectly relevant to the review question as they focused on	High confidence	14 studies contributed, with no or very minor concerns regarding coherence, adequacy and relevance. Although there were minor concerns about methodological limitations, this was only due to 4 studies.

Summary of review finding	Studies (reference) contributing to the review finding	Methodological limitations	Coherence	Adequacy	Relevance	Confidence in the evidence	Explanation of GRADE-CERQual assessment
					broader lifestyle and work-related factors. The studies include a diverse range of occupations (including nurses, midwives, emergency physicians, night-time economy workers, firefighters, and tunneling workers). There were a range of shift types, although 4 studies did not report shift type. 11 of the 14 studies were from the UK or US, although there were 6 different countries in total. Therefore, we had no or very minor		

Summary of review finding	Studies (reference) contributing to the review finding	Methodological limitations	Coherence	Adequacy	Relevance	Confidence in the evidence	Explanation of GRADE-CERQual assessment
					concerns about relevance.		
Many shift workers were aware of the adverse effects of fatigue on their cognitive functioning and decision making, which, in turn, affected their work performance. To compensate for this, they often reported adjusting their behavior by, for example, double-checking their own work and asking colleagues to review their decisions. However, tunnelling workers and an emergency physician believed that fatigue did not negatively impact their work performance.	n=13<br><br>(28-30, 32, 33, 37-39, 41, 42, 51-53)	Minor concerns<br><br>1 study had limitations in its study design, recruitment strategy, and data collection methods. 1 study had limited information on the recruitment strategy. 4 studies did not conduct a sufficiently rigorous analysis. We had minor concerns because these limitations applied to 4 of 13 studies.	No or very minor concerns<br><br>The underlying data directly and unambiguously supported the review finding. There were contradictory data from 2 studies, which suggested that not all shift workers believed that fatigue negatively affected their performance, but this was considered in the review finding's description.	No or very minor concerns<br><br>13 studies contributed to this review finding and represented a variety of examples that illustrated or explained the finding.	No or very minor concerns<br><br>10 of the 13 studies that contributed to the review finding were directly relevant to the review question on shift workers' experiences of sleep disturbance and strategies for managing fatigue, while 3 studies were indirectly relevant to the review question as they focused on broader lifestyle and work-related	High confidence	13 studies contributed, with no or very minor concerns regarding coherence, adequacy and relevance. Although there were minor concerns about methodological limitations, this was only due to 4 studies.

Summary of review finding	Studies (reference) contributing to the review finding	Methodological limitations	Coherence	Adequacy	Relevance	Confidence in the evidence	Explanation of GRADE-CERQual assessment
					factors. The studies include a diverse range of occupations (including nurses, midwives, emergency physicians, emergency service personnel, night-time economy workers, bus drivers, and tunnel workers). There were a range of shift types, although 4 studies did not report shift type. 9 of the 13 studies were from the UK or US, although there were 6 different countries in total. Therefore, we had no or very minor		

Summary of review finding	Studies (reference) contributing to the review finding	Methodological limitations	Coherence	Adequacy	Relevance	Confidence in the evidence	Explanation of GRADE-CERQual assessment
					concerns about relevance.		
Shift workers experienced "*peer pressure to soldier through*" (37) fatigue and believed that they must silently accept and endure fatigue, rather than seek support for it.	n=14<br><br>(28-30, 34, 35, 37, 39, 41, 42, 44, 45, 49, 51, 52)	Minor concerns<br><br>1 study had limitations in its study design, recruitment strategy, and data collection methods. 3 studies had limited information on the recruitment strategy, and 1 of these studies also had limited information on data collection methods. 4 studies did not conduct a sufficiently rigorous analysis. We had minor concerns because these limitations	No or very minor concerns<br><br>The underlying data directly and unambiguously supported the review finding and there were no contradictory data.	No or very minor concerns<br><br>14 studies contributed to this review finding and represented a variety of examples that illustrated or explained the finding.	No or very minor concerns<br><br>11 of the 14 studies that contributed to the review finding were directly relevant to the review question on shift workers' experiences of sleep disturbance and strategies for managing fatigue, while 2 studies were indirectly relevant to the review question as they focused on broader lifestyle and work-related factors, and 1 study was partially	High confidence	14 studies contributed, with no or very minor concerns regarding coherence, adequacy and relevance. Although there were minor concerns about methodological limitations, this was only due to 6 studies.

Summary of review finding	Studies (reference) contributing to the review finding	Methodological limitations	Coherence	Adequacy	Relevance	Confidence in the evidence	Explanation of GRADE-CERQual assessment
		applied to 6 of 14 studies.			relevant as it focused on dietary behaviors. The studies include a diverse range of occupations (including nurses, midwives, emergency physicians, night-time economy workers, bus drivers, flight attendants, and tunnelling workers). There were a range of shift types, although 3 studies did not report shift type. 10 of the 14 studies were from the UK or US, although there were 6 different countries in total. Therefore, we had no or very minor		

Summary of review finding	Studies (reference) contributing to the review finding	Methodological limitations	Coherence	Adequacy	Relevance	Confidence in the evidence	Explanation of GRADE-CERQual assessment
					concerns about relevance.		
The perception of fatigue as normal, combined with the belief that admitting and discussing fatigue shows weakness and a lack of camaraderie, perpetuated a detrimental workplace culture in which fatigue remains unacknowledged and untreated. Shift workers avoided disclosing their fatigue to their employer due to concerns about possible negative repercussions.	n=12<br><br>(28-30, 34, 37, 41, 42, 45, 49, 51-53)	Moderate concerns<br><br>1 study had limitations in its study design, recruitment strategy, data collection methods, and data analysis. 2 other studies did not conduct a sufficiently rigorous analysis and 2 studies had limited information on data analysis. 4 studies had limited information on the recruitment strategy and 2 studies had limited information on data collection methods. We had	No or very minor concerns<br><br>The underlying data directly and unambiguously supported the review finding and there were no contradictory data.	No or very minor concerns<br><br>12 studies contributed to this review finding and represented a variety of examples that illustrated or explained the finding.	No or very minor concerns<br><br>All 12 studies that contributed to the review finding were directly relevant to the review question on shift workers' experiences of sleep disturbance and strategies for managing fatigue. The studies include a diverse range of occupations (including nurses, midwives, night-time economy workers, and bus drivers). There were a range of shift types, although 6 studies	High confidence	12 studies contributed, with no or very minor concerns regarding coherence, adequacy and relevance. Although there were moderate concerns regarding methodological limitations due to 8 studies, the review finding still is a valid representation of the data, in part due to the substantial number of studies contributing to this review finding.

Summary of review finding	Studies (reference) contributing to the review finding	Methodological limitations	Coherence	Adequacy	Relevance	Confidence in the evidence	Explanation of GRADE-CERQual assessment
		moderate concerns because these limitations related to 8 of 12 studies.			did not report shift type. 9 of the 12 studies were from the UK, US or Australia, although there were 6 different countries in total. Therefore, we had no or very minor concerns about relevance.		
Shift workers often reported excessive sleepiness and falling asleep while driving, with some recounting experiences of near-misses and crashes.	n=11 (29, 31, 33-35, 41-43, 48, 50, 52)	Moderate concerns 1 study had limitations in its study design, recruitment strategy, and data collection methods. 3 studies had limited information on the recruitment strategy, and 1 of these studies also had limited information on	No or very minor concerns The underlying data directly and unambiguously supported the review finding and there were no contradictory data.	No or very minor concerns 11 studies contributed to this review finding and represented a variety of examples that illustrated or explained the finding.	Moderate concerns 6 of the 11 studies that contributed to the review finding were directly relevant to the review question on shift workers' experiences of sleep disturbance and strategies for managing fatigue, while 4 studies were indirectly relevant to the	Moderate confidence	11 studies contributed. There were no or very minor concerns regarding coherence and adequacy. However, there were moderate concerns regarding methodological limitations and relevance that weakened the

Summary of review finding	Studies (reference) contributing to the review finding	Methodological limitations	Coherence	Adequacy	Relevance	Confidence in the evidence	Explanation of GRADE-CERQual assessment
		data collection methods. 2 studies did not conduct a sufficiently rigorous analysis. We had moderate concerns, because these limitations related to 6 of 11 studies and weakened the review finding to some extent.			review question as they focused on broader lifestyle and work-related factors, and 1 study was partially relevant as it focused on healthy dietary behaviors. While the studies include midwives, bus drivers, and tunnelling workers, 8 of the 11 studies were in healthcare workers. There were a range of shift types, although 2 studies did not report shift type. 8 of the 11 studies were from the UK or US, and 1 study did not report the country, although there were 4 different countries in total.		review finding in total.

Summary of review finding	Studies (reference) contributing to the review finding	Methodological limitations	Coherence	Adequacy	Relevance	Confidence in the evidence	Explanation of GRADE-CERQual assessment
					Therefore, we had moderate concerns about relevance due to the limited occupational and cross-cultural diversity.		
Shift workers struggled to unwind and mentally detach from work due to rumination and worry, frequently triggered by stressful work events or upcoming shifts, which often made it difficult for sleep to happen naturally and automatically.	n=11<br><br>(32, 35, 37-40, 42, 44, 46, 50, 53)	Moderate concerns<br><br>1 study had limitations in its study design, recruitment strategy, and data collection methods. 1 study had limitations in its study design and data collection methods, with limited information on the recruitment strategy. 3 studies did not conduct a sufficiently rigorous analysis.	No or very minor concerns<br><br>The underlying data directly and unambiguously supported the review finding and there were no contradictory data.	No or very minor concerns<br><br>11 studies contributed to this review finding and represented a variety of examples that illustrated or explained the finding.	Moderate concerns<br><br>5 of the 11 studies that contributed to the review finding were directly relevant to the review question on shift workers' experiences of sleep disturbance and strategies for managing fatigue, while 6 studies were indirectly relevant to the review question as they focused on broader lifestyle	Moderate confidence	11 studies contributed. There were no or very minor concerns regarding coherence and adequacy. However, there were moderate concerns regarding methodological limitations and relevance that weakened the review finding in total.

Summary of review finding	Studies (reference) contributing to the review finding	Methodological limitations	Coherence	Adequacy	Relevance	Confidence in the evidence	Explanation of GRADE-CERQual assessment
		We had moderate concerns because these limitations related to 6 of 11 studies.			and work-related factors. While the studies include midwives, emergency physicians, emergency service personnel, and tunnelling workers, 8 of the 11 studies were in healthcare workers. There were a range of shift types, although 3 studies did not report shift type. 7 of the 11 studies were from the UK or US, although there were 5 different countries in total. Therefore, we had moderate concerns about relevance due to the limited occupational and		

Summary of review finding	Studies (reference) contributing to the review finding	Methodological limitations	Coherence	Adequacy	Relevance	Confidence in the evidence	Explanation of GRADE-CERQual assessment
					cross-cultural diversity.		
Theme 2. Balancing sleep needs with competing responsibilities							
The resulting fatigue from de-prioritizing sleep, and from shift work, adversely impacted the quality of their time with family, leading to adverse emotional impacts and strain on family relationships.	n=14<br><br>(28, 29, 32, 34, 37-42, 44, 52, 53, 55)	Minor concerns<br><br>1 study had limitations in its study design, recruitment strategy, and data collection methods. 4 studies did not conduct a sufficiently rigorous analysis. 3 studies provided limited information on the recruitment strategy. We had minor concerns because these limitations related to 6 of 14 studies.	No or very minor concerns<br><br>The underlying data directly and unambiguously supported the review finding and there were no contradictory data.	No or very minor concerns<br><br>14 studies contributed to this review finding and represented a variety of examples that illustrated or explained the finding.	No or very minor concerns<br><br>10 of the 14 studies that contributed to the review finding were directly relevant to the review question on shift workers' experiences of sleep disturbance and strategies for managing fatigue, while 4 studies were indirectly relevant to the review question as they focused on broader lifestyle and work-related factors. The studies include a	High confidence	14 studies contributed, with no or very minor concerns regarding coherence, adequacy and relevance. Although there were minor concerns about methodological limitations, this was only due to 6 studies.

Summary of review finding	Studies (reference) contributing to the review finding	Methodological limitations	Coherence	Adequacy	Relevance	Confidence in the evidence	Explanation of GRADE-CERQual assessment
					diverse range of occupations (including nurses, midwives, emergency physicians, emergency service personnel, bus drivers, firefighters, and tunnelling workers). There were a range of shift types, although 3 studies did not report shift type. 10 of the 14 studies were from the UK or US, although there were 6 different countries in total. Therefore, we had no or very minor concerns about relevance.		

Summary of review finding	Studies (reference) contributing to the review finding	Methodological limitations	Coherence	Adequacy	Relevance	Confidence in the evidence	Explanation of GRADE-CERQual assessment
Shift workers reported difficulties getting adequate time for sleep between shifts, especially when work schedules did not allow enough time for sleep between shifts.	n=19<br><br>(28, 32, 37, 39, 41, 42, 44, 46, 48, 49, 52, 53)	No or very minor concerns<br><br>1 study had limitations in its study design, recruitment strategy, and data collection methods. 2 studies had limitations in the data analysis. 2 studies provided limited information on the recruitment strategy. Nonetheless, there were only very minor concerns because these limitations applied to only 5 of 12 studies and therefore, did not affect the review finding notably.	Minor concerns<br><br>The majority of data (from 14 studies) directly and unambiguously supported the review finding. However, the 5 studies that mentioned issues of multiple consecutive shifts suggested that working multiple shifts in a row could lead to fatigue, but did not explicitly mention sleep issues. Nonetheless, this does not directly contradict the review finding, and therefore we only	No or very minor concerns<br><br>12 studies contributed to this review finding and represented a variety of examples that illustrated or explained the impacts of shift work schedules on sleep schedules.	No or very minor concerns<br><br>10 of the 12 studies that contributed to the review finding were directly relevant to the review question on shift workers' experiences of sleep disturbance and strategies for managing fatigue, while 2 studies were indirectly relevant to the review question as they focused on broader lifestyle and work-related factors. The studies include a diverse range of occupations (including nurses, emergency	High confidence	19 studies contributed, with no or very minor concerns regarding methodological limitations, adequacy and relevance. Although there were minor concerns about coherence, the majority of the underlying data (from 14 studies) directly and unambiguously supported the review finding.

Summary of review finding	Studies (reference) contributing to the review finding	Methodological limitations	Coherence	Adequacy	Relevance	Confidence in the evidence	Explanation of GRADE-CERQual assessment
			had minor concerns.		physicians, paramedics, bus drivers, and tunnelling workers). There were a range of shift types, although 2 studies did not report shift type. 7 of the 12 studies were from the UK or US, although there were 6 different countries in total. Therefore, we had no or very minor concerns about relevance.		
Shift workers described difficulties balancing daytime sleep with competing responsibilities, including family, work, and leisure. Family responsibilities, including childcare, were particularly	n=18<br><br>(28-30, 34, 37-42, 44, 46-50, 53, 55)	Minor concerns<br><br>2 studies had limitations in the study design, recruitment strategy, and data collection methods. 4 studies	Minor concerns<br><br>The majority of the underlying data (from 15 studies) directly and unambiguously supported the	No or very minor concerns<br><br>18 studies contributed to this review finding and represented a variety of examples that illustrated or	No or very minor concerns<br><br>12 of the 18 studies that contributed to the review finding were directly relevant to the review	High confidence	18 studies contributed, with no or very minor concerns regarding adequacy and relevance. While there were minor

Summary of review finding	Studies (reference) contributing to the review finding	Methodological limitations	Coherence	Adequacy	Relevance	Confidence in the evidence	Explanation of GRADE-CERQual assessment
disruptive to their sleep schedules. Although shift workers recognized the negative impact of insufficient sleep on their health and wellbeing, most prioritized their families' needs over their own sleep. Shift workers with fewer competing responsibilities often had more opportunities for daytime sleep.		did not conduct a sufficiently rigorous analysis. 5 studies provided limited information on the recruitment strategy. 1 study provided limited information on data collection methods. 1 study provided limited information on data analysis. We had minor concerns because these limitations related to 10 of 18 studies.	review finding. However, there were contradictory data from 3 studies, which suggest that some shift workers proactively plan and schedule time with family and sleep, which reduces the negative impacts of shift work. Nonetheless, we had only minor concerns given the amount of studies that were coherent with the review finding.	explained the finding.	question on shift workers' experiences of sleep disturbance and strategies for managing fatigue, while 5 studies were indirectly relevant to the review question as they focused on broader lifestyle and work-related factors, and 1 study was partially relevant as it focused on dietary and exercise behaviors. The studies include a diverse range of occupations (including nurses, midwives, paramedics, emergency physicians, emergency service		concerns regarding methodological limitations and coherence, there were still a substantial number of strong and coherent studies that contributed to the review finding.

Summary of review finding	Studies (reference) contributing to the review finding	Methodological limitations	Coherence	Adequacy	Relevance	Confidence in the evidence	Explanation of GRADE-CERQual assessment
					personnel, night-time economy workers, bus drivers, firefighters, and tunnelling workers). There were a range of shift types, although 5 studies did not report shift type. 12 of the 18 studies were from the UK or US, although there were 6 different countries in total. Therefore, we had no or very minor concerns about relevance given that 12 studies were highly relevant and the diversity in study characteristics.		

Summary of review finding	Studies (reference) contributing to the review finding	Methodological limitations	Coherence	Adequacy	Relevance	Confidence in the evidence	Explanation of GRADE-CERQual assessment
Shift workers reported difficulties getting adequate time for naps during shifts due to inconsistent, late, or an absence of rest breaks. When breaks were available, they were often too short to nap, while some would worry about sleep inertia after napping, leading shift workers to use breaks to relax or eat instead.	n=18<br><br>(30, 31, 33, 35-37, 39, 41-48, 50, 51, 54)	Moderate concerns<br><br>2 studies had limitations in the study design and data collection methods, and 1 of these studies had limitations in its recruitment strategy. 4 studies did not conduct a sufficiently rigorous analysis, and 2 studies provided limited information on data analysis. 3 studies provided limited information on data collection methods. 6 studies provided limited information on the recruitment strategy. We had moderate	No or very minor concerns<br><br>The underlying data directly and unambiguously supported the review finding and there were no contradictory data.	No or very minor concerns<br><br>18 studies contributed to this review finding and represented a variety of examples that illustrated or explained the finding.	Minor concerns<br><br>8 of the 18 studies that contributed to the review finding were directly relevant to the review question on shift workers' experiences of sleep disturbance and strategies for managing fatigue, while 5 studies were indirectly relevant to the review question as they focused on broader lifestyle and work-related factors, and 5 studies were partially relevant as they focused on dietary or exercise behaviors. The studies include a diverse range of	Moderate confidence	18 studies contributed. There were no or very minor concerns regarding coherence and adequacy. However, there were minor concerns regarding relevance, and moderate concerns regarding methodological limitations relating to 11 studies. Altogether, we found that this weakened the review finding, but to a limited extent.

Summary of review finding	Studies (reference) contributing to the review finding	Methodological limitations	Coherence	Adequacy	Relevance	Confidence in the evidence	Explanation of GRADE-CERQual assessment
		concerns because these limitations related to 11 of 18 studies, which contributed substantially to the review finding.			occupations (including nurses, midwives, emergency physicians, paramedics, night-time economy workers, bus drivers, flight attendants, and tunnelling workers). There were a range of shift types, although 5 studies did not report shift type. 9 of the 18 studies were from the UK or US, although there were 7 different countries in total. Therefore, we had minor concerns about relevance.		

Summary of review finding	Studies (reference) contributing to the review finding	Methodological limitations	Coherence	Adequacy	Relevance	Confidence in the evidence	Explanation of GRADE-CERQual assessment
Shift workers reported psychological and emotional consequences from trying to balance sleep with competing responsibilities, such as guilt over missed family time and worrying about failing to meet important responsibilities while sleeping during the day.	n=12<br><br>(29, 32, 34-36, 38, 40, 44, 45, 51-53)	Moderate concerns<br><br>2 studies had limitations in its study design and data collection methods. 4 studies did not conduct a sufficiently rigorous analysis, and 1 study provided limited information on data analysis. 4 studies provided limited information on the recruitment strategy. 1 study provided limited information on data collection methods. We had moderate concerns because these limitations related to 7 of 12 studies, which	No or very minor concerns<br><br>The underlying data directly and unambiguously supported the review finding and there were no contradictory data.	No or very minor concerns<br><br>12 studies contributed to this review finding and represented a variety of examples that illustrated or explained different psychological and emotional consequences.	Moderate concerns<br><br>5 of the 12 studies that contributed to the review finding were directly relevant to the review question on shift workers' experiences of sleep disturbance and strategies for managing fatigue, while 5 studies were indirectly relevant to the review question as they focused on broader lifestyle and work-related factors, and 2 studies were partially relevant as it focused on dietary behaviors. The studies include a diverse	Moderate confidence	12 studies contributed. There were no or very minor concerns regarding coherence and adequacy. However, there were moderate concerns regarding methodological limitations and relevance that weakened the review finding in total.

Summary of review finding	Studies (reference) contributing to the review finding	Methodological limitations	Coherence	Adequacy	Relevance	Confidence in the evidence	Explanation of GRADE-CERQual assessment
		contributed substantially to the review finding.			range of occupations (including nurses, midwives, paramedics, emergency service personnel, and flight attendants), although 8 of the 12 studies were in nurses. There were a range of shift types, although 3 studies did not report shift type. 7 of the 12 studies were from the UK or US, although there were 6 different countries in total. Therefore, we had moderate concerns about relevance due to the limited number of directly relevant studies, as well as limited		

Summary of review finding	Studies (reference) contributing to the review finding	Methodological limitations	Coherence	Adequacy	Relevance	Confidence in the evidence	Explanation of GRADE-CERQual assessment
					occupational and cross-cultural diversity.		
Women found it particularly challenging to balance sleep and family responsibilities alongside their shift work due to gendered expectations.	n=4<br><br>(30, 34, 40, 55)	Minor concerns<br><br>1 study had limited information on data collection methods and data analysis. Another study had limited information on the recruitment strategy. We had minor concerns, because these limitations related to 2 of 4 studies. Additionally, 3 studies did not adequately consider the relationship between researcher and	No or very minor concerns<br><br>The majority of the underlying data (from 3 studies) directly and unambiguously supported the review finding. However, 1 study suggested that male shift workers also feel pressured be both breadwinners and caregivers; while this contradicts the view finding, this was explicitly reported by only one participant, so	Moderate concerns<br><br>Only 4 studies contributed to this review finding. While the studies provide concrete examples and detailed descriptions of how women balancing sleep, family responsibilities, and shift work, and the emotional impacts, there were only 1 study provided sufficiently rich data, with 2	Minor concerns<br><br>3 of the 4 studies that contributed to the review finding were directly relevant to the review question on shift workers' experiences of sleep disturbance and strategies for managing fatigue, while 1 study was indirectly relevant to the review question as they focused on broader lifestyle and work-related factors. The studies include a	Moderate confidence	4 studies contributed. There were no or very minor concerns regarding coherence. However, there were moderate concerns regarding adequacy and minor concerns regarding methodological limitations and relevance. In total, we found that the review finding lost strength, in particular due to

Summary of review finding	Studies (reference) contributing to the review finding	Methodological limitations	Coherence	Adequacy	Relevance	Confidence in the evidence	Explanation of GRADE-CERQual assessment
		participant, which we believe is particularly important for this review finding, and thus weakened the review finding to a limited extent.	we had only very minor concerns.	studies contributing very thin data.	diverse range of occupations and shift types, although shift type was not reported in 1 study. The studies were only from 2 countries (UK and US). Therefore, we had minor concerns about relevance.		the small number of studies.
Some shift workers felt that shift work provided greater flexibility in managing their time, especially those with more control over their hours or those working part-time.	n=9<br><br>(29, 32, 37, 38, 44, 47, 49, 51, 53)	Moderate concerns<br><br>1 study had limitations in its study design, recruitment strategy, and data collection methods 1 study had limited information on data collection methods and data	Minor concerns<br><br>4 studies directly and unambiguously supported the review finding. While the other 5 studies discuss the benefits of specific shift patterns, they do not explicitly link these benefits to	No or very minor concerns<br><br>9 studies contributed to this review finding and represented a variety of examples that illustrated or explained the review finding. The 4 studies that directly contributed to the	Minor concerns<br><br>5 of the 9 studies that contributed to the review finding were directly relevant to the review question on shift workers' experiences of sleep disturbance and strategies for managing fatigue, while 3 studies	Moderate confidence	9 studies contributed, with no or very minor concerns regarding adequacy. However, there were moderate concerns regarding methodological limitations, and minor concerns regarding

Summary of review finding	Studies (reference) contributing to the review finding	Methodological limitations	Coherence	Adequacy	Relevance	Confidence in the evidence	Explanation of GRADE-CERQual assessment
		analysis. 3 studies had limited information on the recruitment strategy. 4 studies did not conduct a sufficiently rigorous analysis. We had moderate concerns, because these limitations related to 5 of 9 studies, and thus weakened the review finding to some extent.	increased flexibility in managing time. Nonetheless, there were no contradictory data, so we only had minor concerns.	review finding (see coherence) provided the largest part of data and highest level of richness to this finding.	were indirectly relevant to the review question as they focused on broader lifestyle and work-related factors, and 1 study was partially relevant as it focused on dietary and exercise behaviors. The studies include a diverse range of occupations (including nurses, midwives, paramedics, and emergency service personnel), although 5 of the 9 studies were in nurses. There were a range of shift types, although 2 studies did not report shift type. 4 of the 9 studies		coherence and relevance. In total, we found that the review finding lost strength, in particular due concerns that methodological limitations.

Summary of review finding	Studies (reference) contributing to the review finding	Methodological limitations	Coherence	Adequacy	Relevance	Confidence in the evidence	Explanation of GRADE-CERQual assessment
					were from the US, although there were 4 different countries in total. Therefore, we had minor concerns about relevance.		
For shift workers with children, support from a partner or extended family was crucial for facilitating sleep by taking on more childcare responsibilities.	n=7<br><br>(28-30, 34, 38-40)	Minor concerns<br><br>1 study had limitations in its study design, recruitment strategy, and data collection methods 2 studies had limited information on the recruitment strategy. 3 studies did not conduct a sufficiently rigorous analysis. We had minor	No or very minor concerns<br><br>The underlying data directly and unambiguously supported the review finding and there were no contradictory data.	Moderate concerns<br><br>7 studies contributed to this review finding and represented a variety of examples that illustrated or explained partner and extended family support for shift workers. However, 4 studies contributed very thin data, with only 2 studies	Minor concerns<br><br>5 of the 7 studies that contributed to the review finding were directly relevant to the review question on shift workers' experiences of sleep disturbance and strategies for managing fatigue, while 2 studies were indirectly relevant to the review question as	Moderate confidence	7 studies contributed, with no or very minor concerns regarding coherence. However, there were minor concerns regarding methodological limitations and relevance, and moderate concerns regarding

Summary of review finding	Studies (reference) contributing to the review finding	Methodological limitations	Coherence	Adequacy	Relevance	Confidence in the evidence	Explanation of GRADE-CERQual assessment
		concerns because these limitations applied to 4 of 9 studies.		contributing adequately rich data.	they focused on broader lifestyle and work-related factors. The studies include a diverse range of occupations (including nurses, midwives, emergency physicians, night-time economy workers, and emergency service personnel). There were a range of shift types, although 4 studies did not report shift type. 5 of the 7 studies were from the US or UK, although there were 4 different countries in total. Therefore, we had		adequacy. In total, we found that the review finding lost strength, in particular due to insufficient rich data.

Summary of review finding	Studies (reference) contributing to the review finding	Methodological limitations	Coherence	Adequacy	Relevance	Confidence in the evidence	Explanation of GRADE-CERQual assessment
					minor concerns about relevance.		
Theme 3. Obstacles to engaging in unhealthy behaviors							
Shift workers preferred organizational interventions, such as scheduled breaks or improved napping facilities, over behavioral interventions. Some shift workers were skeptical about behavioral interventions because they had found them ineffective in the past or believed they were already engaging in healthy behaviors.	n=10<br><br>(28, 29, 32, 33, 37, 39, 48, 49, 51, 53)	No or very minor concerns<br><br>1 study had limitations in its study design, recruitment strategy, and data collection methods. 2 studies had limited information on the recruitment strategy. 3 studies did not conduct a sufficiently rigorous analysis. Nonetheless, there were only very	Minor concerns<br><br>The underlying data directly referred to shift workers preferences for organizational interventions or skeptism about behavioral interventions. However, in 2 studies, shift workers expressed concerns about the challenges of implementing these interventions	No or very minor concerns<br><br>10 studies contributed to this review finding and represented a variety of examples that illustrated or explained the finding.	No or very minor concerns<br><br>8 of the 10 studies that contributed to the review finding were directly relevant to the review question on shift workers' experiences of sleep disturbance and strategies for managing fatigue, while 2 studies were indirectly relevant to the review question as they focused on	High confidence	10 studies contributed, with no or very minor concerns regarding methodological limitations, adequacy and relevance. Although there were minor concerns about coherence, this was only due to 2 studies and there were no directly contradictory data.

Summary of review finding	Studies (reference) contributing to the review finding	Methodological limitations	Coherence	Adequacy	Relevance	Confidence in the evidence	Explanation of GRADE-CERQual assessment
		minor concerns because these limitations applied to only 4 of 10 studies and therefore, did not affect the review finding notably.	in demanding work environments. Nonetheless, no data contradicted their preference for organizational over behavioral interventions.		broader lifestyle and work-related factors. The studies include a diverse range of occupations (including nurses, midwives, emergency physicians, and bus drivers), although 6 of the studies were in nurses. There were a range of shift types, although 2 studies did not report shift type. 5 of the 10 studies were from the US, although there were 6 different countries in total. Therefore, we had no or very minor concerns about relevance.		

Summary of review finding	Studies (reference) contributing to the review finding	Methodological limitations	Coherence	Adequacy	Relevance	Confidence in the evidence	Explanation of GRADE-CERQual assessment
Many shift workers recognized the benefits of napping at work but faced obstacles such as inadequate facilities, insufficient breaks, or lack of managerial support.	n=18<br><br>(28-37, 39, 41, 42, 45, 46, 48, 50, 51)	Moderate concerns<br><br>3 studies had limitations in the study design and data collection methods, and 2 of these studies had limitations in its recruitment strategy. 4 studies had limited information on the recruitment strategy. 3 studies had limited information on the data collection methods. 4 studies did not conduct a sufficiently rigorous analysis, and 2 studies provided limited information on data analysis. We had moderate	No or very minor concerns<br><br>The underlying data directly and unambiguously supported the review finding and there were no contradictory data.	No or very minor concerns<br><br>18 studies contributed to this review finding and represented a variety of examples that illustrated or explained the finding.	No or very minor concerns<br><br>12 of the 18 studies that contributed to the review finding were directly relevant to the review question on shift workers' experiences of sleep disturbance and strategies for managing fatigue, while 4 studies were indirectly relevant to the review question as they focused on broader lifestyle and work-related factors, and 2 studies were partially relevant as they focused on dietary behaviors. The studies	High confidence	18 studies contributed, with no or very minor concerns regarding coherence, adequacy and relevance. Although there were moderate concerns regarding methodological limitations due to 10 studies, the review finding still is a valid representation of the data, in part due to the substantial number of studies contributing to this review finding.

Summary of review finding	Studies (reference) contributing to the review finding	Methodological limitations	Coherence	Adequacy	Relevance	Confidence in the evidence	Explanation of GRADE-CERQual assessment
		concerns, because these limitations related to 10 of 18 studies, and thus weakened the review finding to some extent.			include a diverse range of occupations (including nurses, midwives, paramedics, emergency physicians, night-time economy workers, bus drivers, firefighters, flight attendants, and tunnelling workers). There were a range of shift types, although 5 studies did not report shift type. 11 of the 18 studies were from the UK or US, although there were 7 different countries in total. Therefore, we had no or very minor		

Summary of review finding	Studies (reference) contributing to the review finding	Methodological limitations	Coherence	Adequacy	Relevance	Confidence in the evidence	Explanation of GRADE-CERQual assessment
					concerns about relevance.		
Shift workers reported that high levels of work-related stress and fatigue made it even harder to engage in healthy behaviors. For example, while some shift workers used exercise to manage stress and improve sleep, others felt too tired to exercise after shifts or on days off. Despite efforts to be mindful of their diet, some workers frequently turned to unhealthy food choices as a way to cope with stress and fatigue.	n=9<br><br>(34, 36, 37, 39, 43, 45, 47, 54, 55)	No or very minor concerns<br><br>1 study had limited information on the recruitment strategy. 1 study had limited information on data collection methods. 1 study did not conduct a sufficiently rigorous analysis and 1 study had limited information on data analysis. Nonetheless, there were only very minor concerns because these limitations applied to only 3 of 9 studies and therefore, did not	No or very minor concerns<br><br>The vast majority of underlying data (from 8 studies) directly and unambiguously supported the review finding. In 1 study, some participants highlighted the importance of healthy snacking to maintain energy levels. However, we described this as very minor instead of minor concern, because 8 studies described unhealthy food	No or very minor concerns<br><br>9 studies contributed to this review finding and represented a variety of examples that illustrated or explained the finding.	Moderate concerns<br><br>4 of the 10 studies that contributed to the review finding were directly relevant to the review question on shift workers' experiences of sleep disturbance and strategies for managing fatigue, while 5 studies were only partially relevant as it focused on dietary exercise behaviors. The studies include a diverse range of occupations (including nurses, emergency physicians, flight	High confidence	9 studies contributed. There were no or very minor concerns regarding methodological limitations, coherence and adequacy. Although there were moderate concerns regarding relevance, the review finding was still a valid representation of the data.

Summary of review finding	Studies (reference) contributing to the review finding	Methodological limitations	Coherence	Adequacy	Relevance	Confidence in the evidence	Explanation of GRADE-CERQual assessment
		affect the review finding notably.	choices to manage stress.		attendants, and firefighters), although 5 of the studies were in nurses. There were a range of shift types, although 3 studies did not report shift type. 6 of the 9 studies were from the US or Australia, and 1 study did not report country, although there were 4 different countries in total. Therefore, we had moderate concerns about relevance due to the limited number of directly relevant studies, as well as limited occupational and		

Summary of review finding	Studies (reference) contributing to the review finding	Methodological limitations	Coherence	Adequacy	Relevance	Confidence in the evidence	Explanation of GRADE-CERQual assessment
					cross-cultural diversity.		
Food choices and intake were influenced by erratic work schedules, insufficient breaks, lack of healthy food options, and inadequate facilities at work for storing home-cooked food.	n=12<br><br>(30, 36, 37, 39, 41, 43-48, 54)	Moderate concerns<br><br>1 study had limitations in its study design, recruitment strategy, and data collection methods 2 studies had limited information on the recruitment strategy. 2 studies had limited information on the data collection methods. 2 studies did not conduct a sufficiently rigorous analysis, and 2 studies provided limited information on data analysis. We had minor	No or very minor concerns<br><br>The underlying data directly and unambiguously supported the review finding and there were no contradictory data.	No or very minor concerns<br><br>12 studies contributed to this review finding and represented a variety of examples that illustrated or explained different external barriers related to work schedules and environments.	Minor concerns<br><br>6 of the 12 studies that contributed to the review finding were directly relevant to the review question on shift workers' experiences of sleep disturbance and strategies for managing fatigue, while 1 study was indirectly relevant to the review question as they focused on broader lifestyle and work-related factors, and 5 studies were partially relevant as they focused on dietary or exercise behaviors. The	Moderate confidence	12 studies contributed. There were no or very minor concerns regarding coherence and adequacy. However, there were minor concerns regarding relevance, and moderate concerns regarding methodological limitations. In total, we found that the review finding lost strength, in particular due to methodological limitations related to 7 studies.

Summary of review finding	Studies (reference) contributing to the review finding	Methodological limitations	Coherence	Adequacy	Relevance	Confidence in the evidence	Explanation of GRADE-CERQual assessment
		concerns, because these limitations related to 7 of 12 studies, and thus weakened the review finding some extent.			studies include a diverse range of occupations (including nurses, emergency physicians, paramedics, night-time economy workers, bus drivers, and flight attendants). There were a range of shift types, although 5 studies did not report shift type. 8 of the 12 studies were in the UK or Australia, and 1 study did not report the country, although there were 5 different countries in total. Therefore, we had minor concerns about relevance due to the number of partially relevant		

Summary of review finding	Studies (reference) contributing to the review finding	Methodological limitations	Coherence	Adequacy	Relevance	Confidence in the evidence	Explanation of GRADE-CERQual assessment
					studies, and limited cross-cultural diversity.		
Few shift workers reported receiving sufficient education or training from their organization on how to manage fatigue and improve sleep.	n=3<br><br>(48-50)	No or very minor concerns<br><br>1 study had limited information on the recruitment strategy. Nonetheless, there were only very minor concerns because these limitations applied to only 1 of 3 studies and therefore, did not affect the review finding notably.	No or very minor concerns<br><br>The underlying data directly and unambiguously supported the review finding and there were no contradictory data.	Moderate concerns<br><br>Only 3 studies contributed to this review finding. While the studies provide concrete examples and detailed descriptions of the lack of education and training they receive regarding fatigue management and sleep improvement, and the impacts this has on their ability to manage their sleep and work schedules effectively, the	Minor concerns<br><br>2 of the studies that contributed to the review finding were directly relevant to the review question on shift workers' experiences of sleep disturbance and strategies for managing fatigue, while 1 study was indirectly relevant to the review question as they focused on broader lifestyle and work-related factors. The studies include a diverse range of occupations and shift types. The	Moderate confidence	3 studies contributed. While there were no or very minor concerns regarding methodological limitations and coherence, there were moderate concerns regarding adequacy and minor concerns regarding relevance. In total, we found that the review finding lost strength, in particular due to the small number of studies.

Summary of review finding	Studies (reference) contributing to the review finding	Methodological limitations	Coherence	Adequacy	Relevance	Confidence in the evidence	Explanation of GRADE-CERQual assessment
				data were relatively thin.	studies were only from 3 countries (UK, US and Australia). Therefore, we had minor concerns about relevance.		
Shift workers were aware of actions that would reduce fatigue and benefit their sleep and health, but often struggled to translate this knowledge into their own behavior.	n=14<br><br>(31, 32, 36, 37, 39, 41, 43-48, 50, 54)	Minor concerns<br><br>1 study had limitations in its study design, recruitment strategy, and data collection methods. 3 studies had limited information on the recruitment strategy. 2 studies had limited information on the data collection methods. 3 studies did not conduct a sufficiently rigorous analysis, and 1 study	Moderate concerns<br><br>The majority of underlying data (from 9 studies) directly and unambiguously supported the review finding that shift workers' awareness of actions that would reduce fatigue and benefit their sleep and health. However, there were only 5 studies explicitly discuss the challenges shift workers face	No or very minor concerns<br><br>14 studies contributed to this review finding and represented a variety of examples that illustrated or explained the review finding. The 5 studies that directly contributed to the review finding of the challenges shift workers face in translating knowledge into behavior (see coherence)	Minor concerns<br><br>6 of the 14 studies that contributed to the review finding were directly relevant to the review question on shift workers' experiences of sleep disturbance and strategies for managing fatigue, while 3 studies were indirectly relevant to the review question as they focused on broader lifestyle and work-related factors, and 5	Moderate confidence	14 studies contributed, with no or very minor concerns regarding adequacy. However, there were minor concerns regarding methodological limitations and relevance, and moderate concerns regarding coherence. In total, we found that the review finding lost

Summary of review finding	Studies (reference) contributing to the review finding	Methodological limitations	Coherence	Adequacy	Relevance	Confidence in the evidence	Explanation of GRADE-CERQual assessment
		provided limited information on data analysis. We had minor concerns because these limitations applied to 7 of 14 studies.	in translating the knowledge into behavior, which is a key component of the review finding. Therefore, we had moderate concerns regarding coherence.	provided a high level of richness to this finding.	studies were partially relevant as they focused on dietary or exercise behaviors. The studies include a diverse range of occupations (including nurses, emergency physicians, paramedics, bus drivers, and flight attendants), although 7 of the studies were in nurses. There were a range of shift types, although 4 studies did not report shift type. There were 7 different countries in total, although 1 study did not report the country. Therefore, we had		strength, primarily due to concerns about the coherence of the data, particularly regarding the challenges shift workers face in translating knowledge into behavior.

Summary of review finding	Studies (reference) contributing to the review finding	Methodological limitations	Coherence	Adequacy	Relevance	Confidence in the evidence	Explanation of GRADE-CERQual assessment
					minor concerns about relevance.		
Shift workers relied on behavioral strategies they developed through personal experience over time, emphasizing the importance of discovering strategies that work best for them.	n=3<br><br>(28, 34, 36)	No or very minor concerns<br><br>1 study had limited information on the data collection methods and data analysis. Nonetheless, there were only very minor concerns because these limitations applied to only 1 of 3 studies and therefore, did not affect the review finding notably.	No or very minor concerns<br><br>The underlying data directly and unambiguously supported the review finding and there were no contradictory data.	Serious concerns<br><br>Only 3 studies contributed to this review finding. Further, the data that this finding was based on were superficial and provided limited detail on how the shift workers adapted to shift work based on their own personal experience. Only 1 of the studies stated that all participants in the study held the view that the best strategy to adapt to shift work are	Moderate concerns<br><br>2 of the studies that contributed to the review finding were directly relevant to the review question on shift workers' experiences of sleep disturbance and strategies for managing fatigue, while 1 study was only partially relevant as it focused on dietary behaviors. The studies were only from 3 countries (Iran, US and Australia). There	Low confidence	3 studies contributed. There were no or very minor concerns regarding methodological limitations and coherence. However, there were moderate concerns regarding relevance and serious concerns regarding adequacy. Since there was a small number of studies that contributed to the review finding with limited richness and only included nurses,

Summary of review finding	Studies (reference) contributing to the review finding	Methodological limitations	Coherence	Adequacy	Relevance	Confidence in the evidence	Explanation of GRADE-CERQual assessment
				those which they develop themselves, while the other 2 studies did not quantify the participants representing the according views. We therefore concluded that we had serious concerns about data adequacy.	were rotating and night shifts, although 1 study did not report shift type. The studies only included nurses. Therefore, we had moderate concerns about relevance.		there was a strong weakening of the review finding.
